# Functional Surface Generation by EDM—A Review

**DOI:** 10.3390/mi14010115

**Published:** 2022-12-31

**Authors:** Muhammad Abdun Nafi, Muhammad Pervej Jahan

**Affiliations:** Department of Mechanical and Manufacturing Engineering, Miami University, Oxford, OH 45056, USA

**Keywords:** EDM, electro-discharge coating, surface modification, tribological properties, biocompatibility, corrosion performance

## Abstract

Electro-discharge machining (EDM) removes electrically conductive materials by high frequency spark discharges between the tool electrode and the workpiece in the presence of a dielectric liquid. Being an electrothermal process and with melting and evaporation being the mechanisms of material removal, EDM suffers from migration of materials between the tool and the workpiece. Although unwanted surface modification was considered a challenge in the past for many applications, this inherent nature of the EDM process has recently become of interest to the scientific community. As a result, researchers have been focusing on using the EDM process for surface modification and coating by targeted surface engineering. In order to engineer a surface or generate functional coatings using the electro-discharge process, proper knowledge of the EDM process and science of electro-discharge surface modification must be understood. This paper aims to provide an overview of the electro-discharge surface modification and coating processes, thus assisting the readers on exploring potential applications of EDM-based techniques of surface engineering and coating generation. This review starts with a brief introduction to the EDM process, the physics behind the EDM process, and the science of the surface modification process in EDM. The paper then discusses the reasons and purposes of surface modification and coating practices. The common EDM-based techniques reported in the literature for producing coatings on the surface are discussed with their process mechanisms, important parameters, and design considerations. The characterization techniques used for the analysis of modified surfaces and coating layers, as well as the tribological and surface properties of modified surfaces or coatings are discussed. Some of the important applications of EDM-based surface modification and coating processes are generating surfaces for protective coating, for aesthetic purposes, for enhancing the biocompatibility of implants, for improving corrosion resistance, for improving wear resistance, and for improving tribological performance. The current state of the research in these application areas is discussed with examples. Finally, suggestions are provided on future research directions and innovative potential new applications of the electro-discharge-based surface engineering and coating processes.

## 1. Introduction

### 1.1. Brief Overview of the EDM Process

Electrical Discharge Machining (EDM) is one of the most distinguished non-traditional processes that is used to machine electrically conductive materials, irrespective of hardness or toughness, without any contact between the cutting tool and the workpiece [1,2]. Removal of material in this process occurs by converting spark or electrical discharge energy to thermal energy. Sparking is created by a pulsed generator with the help of two electrodes known as the tool and the workpiece in EDM. The workpiece and tool electrode are submerged into a dielectric fluid. Initially, an open gap voltage (40–100 V) is applied to create an electric field [3]. A servo system is used to decrease the gap between the electrodes. As the distance between the electrodes decreases, the intensity of the electric field increases. When the gap is in the range of 0.01–0.5 mm, the electric field intensity surpasses the dielectric strength [4]. As a result, the dielectric fluid is ionized, and an enormous number of ions pass through the dielectric medium from the tool to the opposite pole or workpiece. These vast ions collide with the workpiece at a high speed that is visualized as spark. The distance between the electrodes at which the sparking occurs is known as the sparking gap. A very high heat is produced in the sparking channel, which raises the temperature ranging from 6000 °C to 12,000 °C [5]. The heat generated in this process instantly melts and vaporizes the workpiece regardless of the strength and hardness of the material [1]. The spark energy determines the amount of material that is to be removed from the workpiece. The voltage as well as the resistance rise with the decrease in current due to the disorder of ionized particles. At one point, the current is turned off, and the new dielectric fluid is drawn into the spark gap to wash away the debris [6].

EDM is mainly classified based on the shape and motion of the tool electrode. Basically, there are two types of EDM: die-sinking EDM and wire-EDM. The basic principle of both EDM types is the same. In die-sinking EDM, the tool electrode is shaped in such a way that it produces the mirror image on the workpiece surface. On the other hand, in case of wire-EDM, a wire is used as the tool electrode, which is operated by a CNC machine. The servo controller determines the distance between the electrodes; it plays a vital role in determining surface integrity by ensuring stable machining. There are two types of servo controllers used in the EDM process: electric and hydraulic [7]. Electric servo controllers are used for lightweight machines, whereas hydraulic servo controllers are used in larger machines. The pulse generator is the prime component of any kind of EDM machine. There are two types of pulse generators: the transistor type, which is used widely due to its higher material removal rate; and the resistor-capacitor (RC) type, which is used in the case of machining micro-parts [8]. However, the process of micro-EDM is identical to the conventional EDM types, where a very small amount of energy is needed for each discharge in order to maintain precision and better surface quality, and the axes resolution is in micro scale with a sub-micron resolution.

Various performance parameters are used in order to evaluate the machinability of the EDM process. The material removal rate (MRR) is known as the amount of material removed from the workpiece per unit of time. The tool wear rate (TWR) is the amount of tool eroded from the tool per unit of machining time. A lower value of TWR is expected as a higher TWR results in the frequent change of the tool electrode, which further increases the overall production cost. The electrode wear ratio (EWR) is the ratio of the TWR to the MRR. The surface roughness or more commonly the average surface roughness (R_a_) is the measurement of the machined surface finish after machining and often measured in terms of micrometers (µm). The mentioned performance parameters depend on the process parameters of EDM, which fall into two major categories: electrical and non-electrical parameters. The electrical parameters include the discharge energy, open voltage, gap voltage, peak current, pulse duration, pulse interval, duty ratio, etc., whereas the non-electrical parameters can be related to the electrode and wire parameters for die-sinking and wire-EDM, respectively.

The process parameters mainly define the performance related to the EDM process. The electrical parameters can be controlled by the pulse generator. The discharge energy is the amount of energy supplied per pulse for EDM and plays a significant role in determining the EDM performance. The gap voltage is the voltage applied between the electrodes in EDM. The discharge voltage is the voltage at which the discharge between the electrodes occurs. A higher voltage and current result in higher discharge energy. As a result, more material is removed from the workpiece and the MRR increases; however, it also increases the TWR. The peak current is the highest amount of electricity provided during the machining process. The pulse on-time and pulse off-time are defined by the duration of time when voltage is applied and not applied, respectively. A lower pulse off-time or pulse interval results in a short circuit. The spark energy is controlled by the peak current along with pulse on-time [9], whereas pulse on-time together with open gap voltage are the most significant parameters for determining the MRR [10].

**Figure 1:**
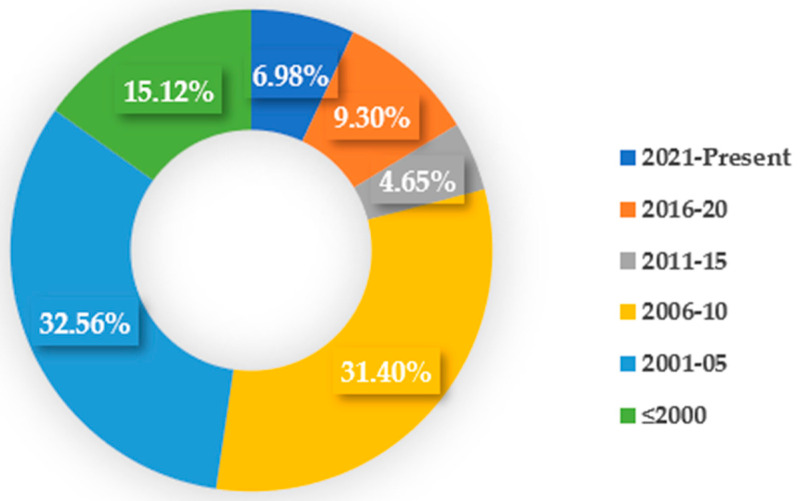
Number of papers considered in this study with publication years.

The surface generation during the EDM process has long been a subject of interest to the non-traditional manufacturing research community. Being an electro-thermal process, the surface generated during the EDM process suffers from various surface and sub-surface defects, and the surface undergoes surface modification due to the migration of materials from the tool electrode and dielectric medium. Although researchers working on the EDM area initially focused for the most part on minimizing the surface modification as much as possible to minimize the alteration of mechanical properties and functionality of the machined surface, a recent research trend has focused on how to utilize those modified surfaces for functional applications. Researchers have even focused on targeted modification of the surface by a variety of techniques so that the modified surface can be used for functional applications. Targeted elements from the tool electrode and dielectric can be deposited on the machined surfaces due to oxidation and carbonization processes happening during the EDM process. Some studies have even focused on using EDM as a coating process to provide protective coating layers on the machined surface. Others have focused on making the surface biocompatible or resistant to corrosion, wear, and other mechanical or environmental extreme conditions. In this regard, this study aims to provide a comprehensive overview of the EDM-based surface modification techniques reported in the literature for making functional surfaces. The generation of subsurface or coating can be achieved by a single step using EDM whereas multiple steps are required in the case of other manufacturing processes. A total of 86 papers related to EDM-based surface modification processes were taken into account for several analyses. Figure 1 shows the number of papers from different publication years that were considered in this study.

### 1.2. How the Surface Modification Happens Naturally in the EDM Process

Primarily, the main purpose of the EDM process is to machine electrically conductive materials possessing high hardness that are difficult to machine using traditional machining processes. It was observed that surface modification naturally occurs during the EDM process due to high heat generation, which causes the melting and evaporation of materials. A higher MRR results in a lower machining time; however, it also affects the surface quality of the machined workpiece [11]. As a result, the parameters associated with a higher value of MRR are also responsible for higher surface roughness and surface modification. The amount and size of craters within the surface increase with the increase in the peak current and pulse on-time. During the EDM process, the workpiece material melts and vaporizes due to the high thermal energy associated with the process. Nevertheless, a small number of vaporized materials resolidify during the pulse off-time period [12]. Therefore, a higher pulse interval results in a higher re-solidification rate of melted materials. The tool electrode melts due to the high amount of heat produced, a fraction of which also solidifies over the workpiece surface. So, the formation of new layers or an altered surface composition is inevitable in the EDM process. The electro-discharge machined surface layers can be divided into three distinctive layers: the spattered EDM surface layer, recast layer, and heat affected zone [13]. All three layers combined indicate the EDM-modified surface above the base material.

The topmost layer is called the spattered layer. This layer is the result of the re-solidification of the molten tool and workpiece materials, which form spatters on the surface. Additionally, this layer contains a high amount of carbon, which is the outcome of the breakdown of the dielectric fluid. The spattered layer can be easily removed by subsequent machining. The layer beneath the spattered layer is the recast or white layer.

**Figure 2:**
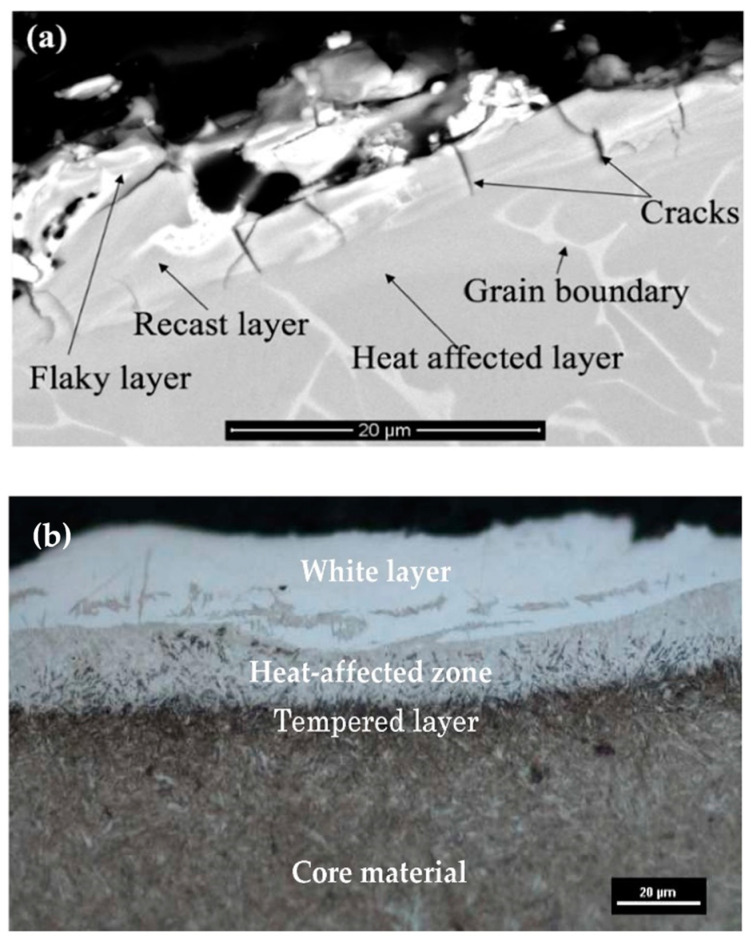
The layers formed over (**a**) Ti-6Al-4V [14] and (**b**) 55NiCrMoV7 [15] after EDM processing. (With kind permission from the respective publications).

This layer forms because of the insertion of molten materials onto the craters. The heat affected zone (HAZ), also known as the annealed layer, is situated below the recast layer. This is the part of base material which has experienced high heating. Figure 2 shows various layers that were produced after the EDM process on the surface of two different alloys.

Due to the rapid heating and melting involved in the EDM process, micro-cracks also develop in the altered metal zone [16]. Surface cracks develop in the recast layer and ends before reaching the heat affected zone. Penetrating cracks are the cracks that penetrate the white layers and further ends in the base material. These kinds of cracks can result in the failure of the machined workpiece [17,18]. Because of unavoidable surface modification involved in the ordinary EDM process, researchers have been working on modifying the EDMed surface in such a way that it possesses better mechanical, electrical, as well as thermal properties. The amount of gross material addition onto the surface can be increased by varying the input parameters [19,20], using composite electrodes [20,21,22], and using powder-mixed dielectric [19,23,24] during EDM.

## 2. Purpose of Surface Generation/Modification/Coating

### 2.1. To Enhance Surface Properties

To be used in extreme environmental and industrial conditions, the materials and alloys developed nowadays need to have better mechanical and thermal properties [25]. One of the main purposes of surface modification or coating is to enhance the surface properties of the materials. The white layer produced during EDM is a topic of interest to the researchers as this layer cannot be easily removed from the workpiece surface. Furthermore, this layer is further hardened after machining [26]. As a result, this layer possesses high hardness and brittleness.

Rebelo et al. [27] used copper electrode and hydrocarbon dielectric fluid to machine different kinds of steel surfaces. The carbon content was found at the machined surface because of the pyrolysis of the hydrocarbon dielectric, which was nine times greater than the base material. The intensity of the carbon percentage is the highest on the machined surface and sharply reduced with the increase in the depth from the machined surface. The carbon percentage also decreased with the decrease in pulse energy. It was found that the recast layer thickness depends on the pulse duration and pulse current [28]. A similar trend of hardness was established when aluminum underwent EDC processing [29]. Jahan et al. [30] demonstrated an increased micro-hardness of Ti-6Al-4V and NiTi alloy surfaces after micro-EDM processing.

Kashaev et al. [31] compared the fatigue performance of electro-discharge-modified Inconel 625, Inconel 718, and Ti-6Al-4V micro-specimens. It was observed that the Ti-6Al-4V provided the best result among the three specimens when tested until fatigue failure. Klocke et al. [32] machined the surface of Ti-6Al-4V by grinding and wire-EDM and compared the specimens in order to investigate the fatigue performance. Though the ground specimen had a lower surface roughness than the eroded specimen, the fine finished eroded specimen came out with a better result in the fatigue performance at the 10%, 50%, and 90% failure probability.

### 2.2. To Enhance Biocompatibility and Corrosion Resistance of the Surface

Replacement of a joint is a common orthopedic surgery that is conducted all over the world. The biocompatibility of implants is a prime concern, which is determined by the surface quality and chemical composition of the material to be implanted. The surface must be non-toxic so that it does not result in any allergy or inflammation inside the host body [33]. The mechanical properties of the implant materials are also required to be almost similar to the properties of human bones, such as in terms of high fatigue strength and a low Young’s modulus.

Titanium alloys have been a popular choice for implant materials because of their high specific strength, corrosion resistance, wear resistance, and biocompatibility. However, titanium alloys are not directly used in the human body without being treated due to the high modulus of elasticity and low fatigue endurance, which can result in the loosening of the implant [34]. Prakash et al. [35] investigated the surface of β-phase Ti alloy by powder-mixed EDM processing using silicon powder in the dielectric fluid, which resulted in a smooth surface with a higher MRR. The recast layer, which was formed on the top of the workpiece, was thinner and well attached. The fatigue endurance limit was higher (250 MPa) on the surface obtained by powder-mixed EDM due to lower surface defects.

Coating over the implant surface can play a significant role in introducing body cells to grow over the coated implant surface and can play a role in repelling bacterial growth; however, this involves at least two distinct processes [36,37,38]. In comparison to that, EDM can provide a single-step solution. Bui et al. [39] investigated the antibacterial properties of a machined surface of Ti-6Al-4V by mixing silver nanoparticles with the dielectric fluid; the authors reported improved resistance to bacteria cultures due to silver content on the machined surface.

**Figure 3:**
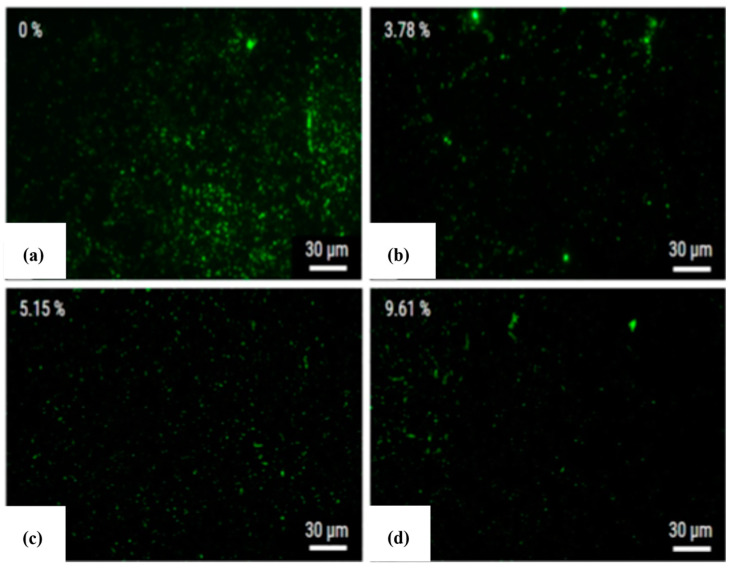
Amount of *S. aureus* bacteria on a Ti-6Al-4V surface, machined using (**a**) 0%, (**b**) 3.78%, (**c**) 5.15%, and (**d**) 9.61% silver on the dielectric fluid [39]. (With kind permission from Elsevier).

A coating layer containing elements from electrodes, powder particles, and dielectric fluid was formed onto the alloy surface. The green marks on Figure 3 represent the *S. aureus*, which was cultivated for 1 day onto the surface machined by PMEDM. The machined surface clearly demonstrated resistance to bacteria and bacteria clusters at 3.78% silver content. The silver percentage at the surface increased with the increase in silver powder in the dielectric fluid. The presence of silver was not only found at the machined surface, but also at the recast layer formed.

**Figure 4:**
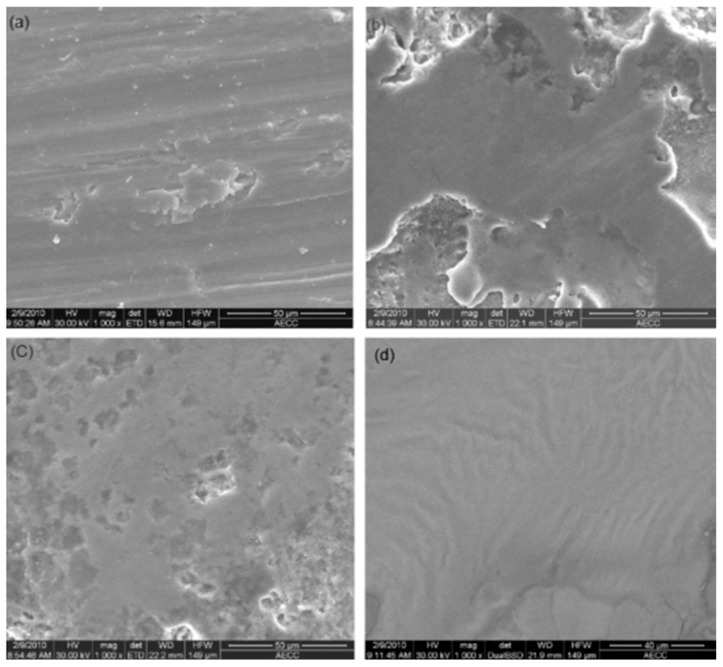
Scars produced due to corrosion in the (**a**) Ti-6Al-4V alloy, (**b**) ESA in air, (**c**) ESA in N_2_, and (**d**) ESA in silicone oil [44]. (With kind permission from Elsevier).

The materials can corrode inside the body when used as an implant due to motion, plaque formation, and irregular cleanliness, which results in releasing harmful ions into the body fluid [40,41,42]. The corrosion performance of the implant material can significantly increase by generating a layer or modifying surface using EDM [43]. Bin et al. [44] investigated the feasibility of enhancing the corrosion resistance of a titanium (Ti) alloy surface machined by using three different dielectrics and a graphite electrode in an EDM process. The surface machined by using air as the dielectric fluid had the poorest corrosion resistance, even from the original Ti alloy, which is shown in Figure 4. The surface obtained using air as a dielectric becomes more prone to corrosion because of the poor surface quality, consisting of micro-cracks and pores. The machined surface layer generated by nitrogen consisted of fewer micro-cracks and better corrosion resistance than the previous one. The workpiece that was machined by using silicone oil as the dielectric provided the best corrosion resistance result. Sharma et al. [45] performed the corrosion test on a coated Ti alloy, which was prepared by using boron nitride powder. The coated alloy came out with a better result than the parent alloy in both stagnant and flowing water conditions. The corrosion rate was lower on the coated alloy due to the formation of TiN and TiAlN during the EDC process.

Toshimitsu et al. [46] investigated the modification of the surface of SKD 11 steel by using chromium powder with kerosene dielectric fluid. Copper was used as the electrode in this investigation, and the corrosion test was conducted using 1% NaCl solution. It was found that the corrosion resistance remarkably increased due to the formation of a uniform layer over the workpiece containing chromium. The same SKD 11 alloy was coated using silicon electrode by Sumi et al. [47]. An amorphous silicon layer of about 10 µm was formed over the surface by this electro-discharge coating (EDC) process. An aqua regia dip test was performed to investigate the corrosion resistance of the coated workpiece. It was observed that the area of the workpiece that contained the Si layer was not damaged in this test, meaning that this layer was not corroded at all. On the other hand, the area which did not contain the coated layer experienced a 0.15 mm corrosion. The grain boundary was visible in this case, and a bubble was formed during the EDM process.

## 3. Electro-Discharge-Based Surface Modification and Coating Techniques

**Figure 5:**
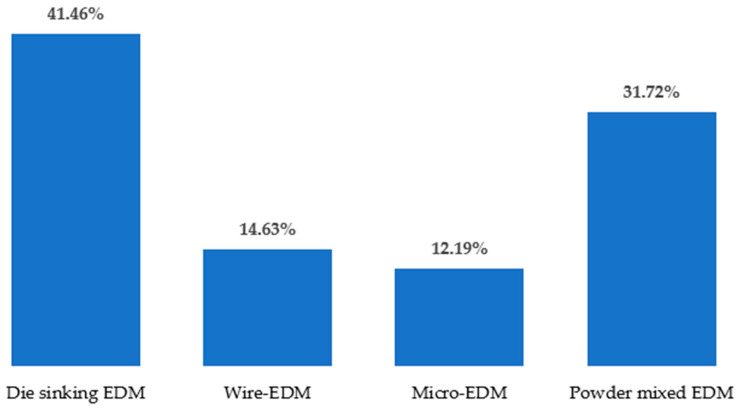
Types of EDM processes reviewed in this study.

**Figure 6:**
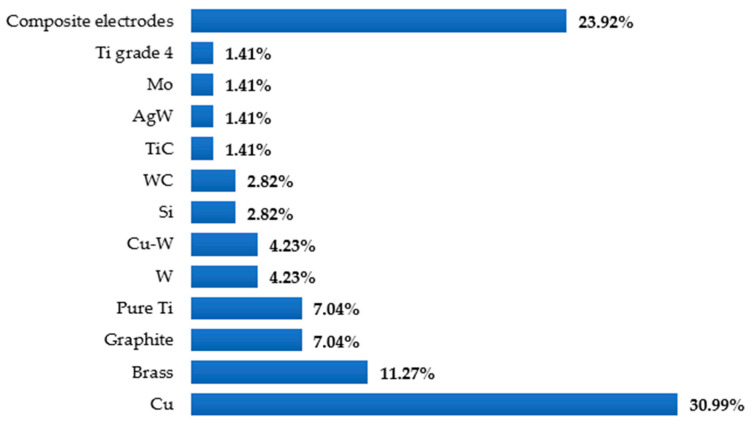
Types of electrodes used in different EDM processes.

Different types of EDM processes have been used by researchers over the years to modify the surfaces of various materials as well as alloys that are electrically conductive. One of the advantages of the mentioned process is that no additional machines are required to coat the surface of the workpiece. In addition, the surface modification occurs almost naturally during the EDM process without special arrangement. The surface engineering using the EDM process does not require expensive equipment, corrosive liquid, or an inert environment. The EDM process itself is advantageous over other machining processes because of the minimum-to-no cutting forces, lesser cost associated with the cutting tools, and capability of machining some of the hardest and most difficult-to-cut materials. Figure 5 shows different types of EDM processes, whereas Figure 6 shows the variations in the electrodes that were considered to conduct this study. It is clear from this analysis that die-sinking EDM is mostly used by researchers, followed by other EDM processes. On the other hand, copper (Cu) is popular among researchers and is frequently used as a tool electrode due to its availability and high conductivity.

### 3.1. Traditional EDM Processes: Die-Sinking and Wire-EDM

Die-sinking and wire-EDM are two conventional EDM processes. The main difference between these two machining processes is the workpiece, electrode arrangement, and target applications. In wire-EDM, a metallic wire is used as the tool electrode, which is guided with the help of a wire drive system and automated wire threading. In die-sinking EDM processing, the tool electrode creates a mirror image of the tool electrode over the workpiece surface.

Due to high heat generation during the EDM processing, not only the workpiece but also the tool electrode experiences melting. As a result, materials from the tool electrode along with the workpiece debris accumulate over the workpiece surface. Numerous studies have been conducted to investigate the layers generated over the workpiece surface by EDM. The composition of the newly formed surface changes with the change in the tool electrode and dielectric fluid.

Arooj et al. [48] found the presence of Cu, O, and C along with the base materials at the newly generated surface when Al 6061 T6 (an alloy of Al that contains Mg and Si) was machined by using a Cu electrode in the presence of an industrial grade kerosene oil as the dielectric fluid during die-sinking EDM. The presence of C was noticeable at the recast layer when Al 2024 T6 alloy was machined using the same tool electrode and dielectric in another study [49].

Various tool electrodes, namely graphite, electrolytic Cu, and Al were used along with kerosene oil as a dielectric to machine titanium grade 5 alloy in die-sinking EDM [50]. The recast layer thickness was the highest in the case of the graphite tool electrode. The Al tool electrode had the highest wear rate during machining, which further increases with the increase in pulse current and pulse duration. The surface cracks can be reduced with the decrease in pulse current in the case of Al and graphite electrode; however, these cracks were omnipresent in the case of the Cu electrode. In another study, this alloy was reported to be machined by a wire-EDM process using a brass wire and deionized water as the tool electrode and dielectric fluid, respectively [43]. Cu and Zn were found on the newly formed layer over the base metal, and the percentages of these elements were found to increase on the smoother surfaces. Various compositions of titanium oxides were found on the white layer.

Duplex stainless steel was machined by die-sinking EDM using graphite, Cu-W, and W electrodes in different dielectric fluids [51]. A thick white layer was produced that contained significant amounts of oxygen, oxides of different elements, and tungsten carbide. Another study machined 316L stainless steel by using a graphite tool electrode in a paraffin oil dielectric medium [52]. Two distinct layers were formed over the base material; the outer layer was dendritic in nature, which was produced by the accumulation of removed material during EDM processing, and the inner layer was primarily a recast layer that was followed by dendrites. The amount of carbon varied in each layer, whereas chromium and iron were present in significant amounts in all layers.

### 3.2. Powder-Mixed EDM

In powder-mixed EDM processing, electrically conductive, semi-conductive, or abrasive powder particles or powder are added with the dielectric fluid with the goal of modifying the machined surface in addition to improved performance. The powder particles are often targeted to be deposited on the machined surface. The addition of electrically conductive powder significantly increases the conductivity level of the dielectric fluid. Moreover, the spark gap also increases due to the lower insulation strength of the dielectric fluid. So, the debris can be easily flushed out from the sparking gap, resulting in a better surface finish of the workpiece [24].

**Figure 7:**
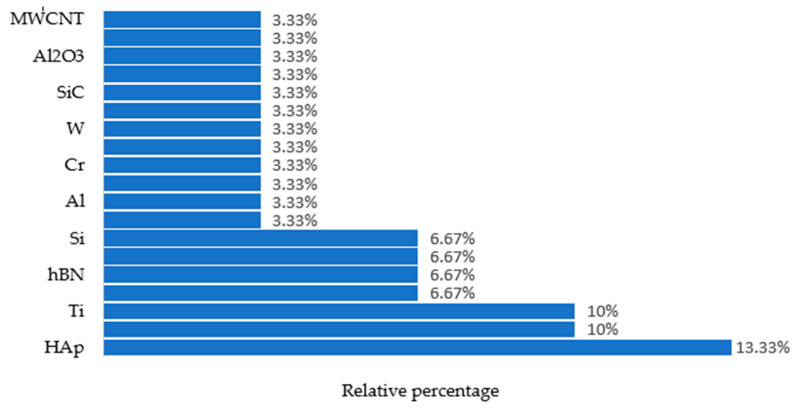
Types of powders used in different EDM processes covered in this study.

Figure 7 shows the use of different powders in various EDM processes that were taken into consideration during this study.

**Figure 8:**
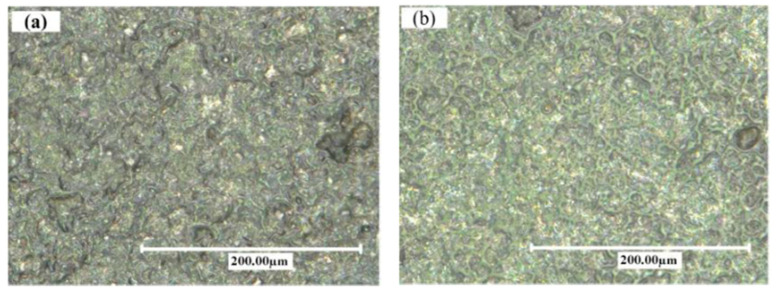
Surfaces of machined SiCp/Al composite obtained by (**a**) conventional EDM and (**b**) powder-mixed EDM [53]. (With kind permission from Elsevier).

Hu et al. [53] compared the machined surfaces of SiCp/Al composite obtained by conventional EDM and powder-mixed EDM processes, which are shown in Figure 8. Aluminum powder was mixed with the kerosene dielectric fluid during the powder-mixed EDM. The average surface roughness values were 0.834 µm and 0.571 µm for conventional EDM and powder-mixed EDM, respectively. The percentages of Al and Si were higher in the powder-mixed EDMed surface. Aluminum powder increased the conductivity of the working fluid, which resulted in an increased sparking gap.

The working fluid easily broke down in the presence of powder-mixed dielectric and the discharge time increased at a constant current and pulse width. As a result, the Si percentage increased during the powder-mixed EDM process. The surface roughness of powder-mixed EDMed surface was 31.5% lower than the machined surface obtained from conventional EDM. The conventional EDMed surface consisted of a higher number of cracks and holes due to the higher discharge energy and short circuiting involved in this process. The inter-electrode capacitance effect during conventional EDM resulted in the distribution of irregular and larger shapes of pits. On the other hand, powder-mixed EDM produced a smoother surface with lesser defects. The formation of micro-cracks largely depends on the discharge energies used during the EDM process and effective discharge energy levels at the gap. As the effective discharge energy involved in the powder-mixed EDM process is lower, there was a lower number of cracks on the powder-mixed EDMed surface. The surface micro hardness was 40% higher in the case of the powder-mixed EDM compared with the conventional EDM process.

**Figure 9:**
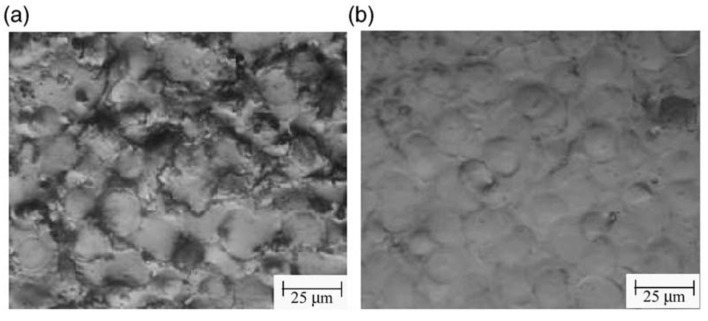
Variation of machined H13 surfaces achieved by (**a**) conventional EDM process and (**b**) powder-mixed EDM by using electrodes with an area of 32 cm^2^ [54]. (With kind permission from Elsevier).

Pecas and Henriques [54] investigated the effectiveness of mixing silicon powder with dielectric fluid to modify the surface of AISI H13 steel (shown in Figure 9). They found that the surface quality was significantly improved in the case of the powder-mixed EDM process compared with the traditional EDM process. Moreover, the presence of powder in the dielectric formed smaller craters on the surface of the workpiece, which played a vital role in producing a smoother surface. They also found from further investigation that the surface finish could be improved with the increase in polishing time as the irregular craters could be removed from the machined surface.

**Figure 10:**
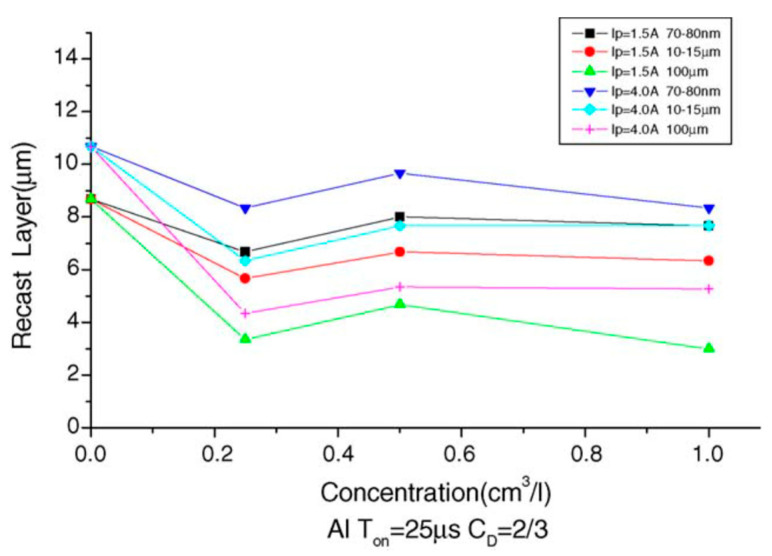
The change in the recast layer thickness with the change in aluminum powder particle size and powder concentration [55]. (With kind permission from Elsevier).

Yih-fong and Fu-chen [55] machined the surface of SKD 11 steel using powder-mixed EDM and found that the size of powder particles played a significant role in determining the surface quality. The roughness of the surface depended more on the particle size of the powder than the powder concentration. Among the powder used, the aluminum powder was found to be the best one in decreasing the surface roughness due to its excellent electrical and thermal properties. Additionally, they found that the recast layer thickness decreased when the powder was introduced in the dielectric. The variation of the newly formed recast layer thickness with the concentration of the Al powder is shown in Figure 10. The particle size was an important factor determining the recast layer thickness. The recast layer thickness increased with the decrease in particle size. The aluminum powder resulted in the thinnest recast layer, whereas the copper powder resulted in the thickest recast layer.

Al-Amin et al. [56] processed a 316L steel surface by introducing multiple additives in the mineral oil dielectric. Carbon nanotubes (CNT) powder with a concentration of 0.5 g/L was added with hydroxyapatite powder (HAp), and pure Ti was used as the cutting tool electrode. The recast layer thickness decreased from 15.29 µm to 12.4 µm when CNT powder was introduced along with HA powder. Additionally, the surface roughness significantly decreased from 4.08 µm to 3.16 µm after using the CNT powder-mixed dielectric.

Other than the powder particle size, powder materials have a great effect on the performance of powder-mixed EDM. Rajesh et al. [57] investigated the surface of AISI 304 steel after PMEDM processing. The particle size of MoS_2_ (40 µm and 90 nm) and duty factor (2, 6, 10) were considered as the input parameters. Ultrasonic vibration was adopted for better flushing, whereas parameters such as the peak current, discharge duration, gap voltage, and powder concentration were constant throughout the experiment. It was found that MoS_2_ was deposited on the machined AISI 304 steel surface when the powder particle size was 90 nm at a lower duty factor, and the recast lower thickness was 14.02 µm. Deeper craters were observed at the 40 µm particle size, and shallower craters were found at the 90 nm particle size at a constant duty factor. Singh et al. [58] investigated graphite powder-mixed dielectric fluid to optimize surface the micro-hardness and surface finish of superalloy Super Co 605. They used a cylindrical graphite piece as the electrode. A modified dielectric fluid flow system was used so that the conductive graphite powder could not contaminate the whole dielectric EDM oil. A noticeable amount of powder was deposited onto the newly formed surface along with the increase in carbon percentage. Additionally, the surface roughness was lowered in the case of powder-mixed EDM with the decrease in crater development. Sharma et al. [59] reported that the melting point of the powder additive had an effect on the surface roughness. They used zirconium and manganese powder-mixed dielectric fluid and found that zirconium powder resulted in a lower surface roughness (SR).

The manganese powder had a lower melting point, so it had a higher evaporation rate. As a result, the deposition of manganese is lower on the machined surface.

Hosni and Lajis [60] used chromium powder with the dielectric in order to machine the surface of AISI D2 hardened steel. The use of chromium powder resulted in a dramatic reduction in the recast layer thickness. The recast layer thickness further decreased when nano-chromium powder was used instead of micro-chromium powder, indicating the effectiveness of nano-scale powders in powder-mixed EDM.

### 3.3. Micro-EDM

Micro-machining is a newer trend compared with conventional machining processes that focuses on fabricating final products with at least one dimension in the micrometer range. Micro-EDM is the micro-scale version of the conventional EDM process. Compared with conventional EDM, micro-EDM produces a smoother surface when small energy at a high frequency is provided for each discharge [3].

Jahan et al. [61] used die-sinking micro-EDM to machine tungsten carbide by different electrodes. The smoothest nano-surface was obtained when the workpiece was machined by silver tungsten (AgW) electrode. The lowest amount of carbon at the newly created surface was found in the case of the AgW tool electrode. A comparative study between die-sinking and milling micro-EDM was performed in another study by the same authors while mixing graphite nano-powder with the dielectric fluid [62]. The machining was performed using a W electrode. The accumulation of carbon over the generated layer increased with the increase in powder concentration in the case of sinking micro-EDM, whereas in the case of milling micro-EDM, the percentage of carbon over the newly formed surface was lower. The craters were homogenously distributed over the recast layer in the case of milling micro-EDM. It was found that the surface roughness in both cases was reduced with the increase in powder concentration up to a certain level. However, a higher powder concentration was found to cause deposition of powder particles on the machined surface.

Davis et al. [63] investigated powder-mixed EDM processing by mixing zinc powder with the dielectric fluid to machine Nitinol using different electrodes. Oxides and carbides were formed over the recast layer, which tended to increase surface micro-hardness. Furthermore, elements from the electrodes were also deposited onto the white layer.

### 3.4. EDM with Composite Electrodes

The selection of an electrode plays a significant role in determining the quality of the surface produced in the EDM process. During the EDM process, the materials migrate from the tool electrode and dielectric to the workpiece, thus modifying the machined surface of the workpiece. Moreover, the performance of the electrodes relies upon the mechanical, thermal, and electrical properties of the electrode. Numerous researchers have investigated the feasibility of using composite electrodes to generate new layers over the workpiece surface rather than using metal electrodes in EDM.

Tsai et al. [64] machined the surface of AISI 1045 medium carbon steel with Cu-Cr composite electrode and Cu metal electrode. Cu and Cr particles moved from the electrode to the workpiece and adhered to it when a negative polarity was used for machining. The surface produced using the Cu electrode had a lower surface roughness. The recast layer was found thinner when Cu-Cr was used as the electrode to machine the workpiece. On the other hand, cracks and pores were found in the recast layer of the workpiece machined by the Cu electrode. Senthilkumar and Reddy [65] used Cu-40% B_4_C electrode to machine a mild steel surface and further compared it with the surface machined by a Cu metal electrode. The Cu-B_4_C electrode was prepared by the powder-metallurgy route. No element transfer occurred from the electrode to the workpiece when the metal electrode was adopted. Additionally, a thin layer of carbon was formed due to the dielectric breakdown. B, B_4_C, and Cu migrated from the electrode to the base material to form BFe_2_, B_4_C, and CuB_28_ layers when mild steel was machined using a composite electrode. Wang et al. [20] used a Ti powder green compact electrode in order to machine the carbon steel surface by conventional EDM processing. A 20 µm titanium carbide (TiC) ceramic layer was found after machining the workpiece surface. The percentage of Fe decreased, and the percentage of TiC increased from the base material to the surface. No specific boundary between the TiC layer and the parent material was found, which was the main reason of the increased hardness of the newly formed layer compared with the base material. W and Cu powders were mixed at 25% and 75% by weight, respectively, in order to prepare sintered compact tools to produce a surface over C-40 grade steel [66]. The newly formed surface thickness varied from 3 to 785 µm, which was mainly formed by W and its carbides. The percentage of Fe was lower at the top surface. In another study, 60% WC and 40% Cu (by weight) were mixed to prepare a P/M compact tool electrode to generate a new layer over the same workpiece [67]. A successful deposition of WC occurred in this experiment, and the newly formed layer contained various carbides of W.

Li et al. [68] used a Cu-SiC composite electrode to modify the surface of titanium grade 5 alloy and compared the prepared surface with another surface of the same alloy that was prepared by using a Cu electrode. The recast layer thickness decreased in the case of the Cu-SiC electrode, which was formed due to the migration of Cu and Si from the electrode along with base material and C from the dielectric. This surface is also responsible for significantly increasing the hardness due to the formation of TiC and TiSi_2_.

Sarmah et al. [69] mixed equal weight of Inconel 718 and Al powder to prepare green compact electrode in order to generate a surface over Al 7075 alloy using die-sinking EDM. The machined surface contained deposited layer, globules, voids, craters, and microcracks. The EDM involved melting and vaporization followed by a rapid cooling of molten material. Furthermore, the molten material did not flush away due to improper flushing. As a result, the unflushed molten material cooled down and produced a recast layer over the base material and globules in addition to microcracks, craters, and voids due to the residual stress. It was also found that globules were of different shapes and sizes. Smaller globules were the result of recondensed material from the vapor phase and larger globules were due to the incomplete or partial evaporation of eroded material. Partially evaporated globules produced a smoother surface, whereas recondensed globules resulted in a flaky surface.

Generation of new layers by various kinds of EDM processes are shown in Table 1.

## 4. Characterization Techniques Used to Investigate Surface and Sub-Surface Modification by EDM

### 4.1. Tribo-Testing of EDMed Surface for Tribological Chracterization

**Figure 11:**
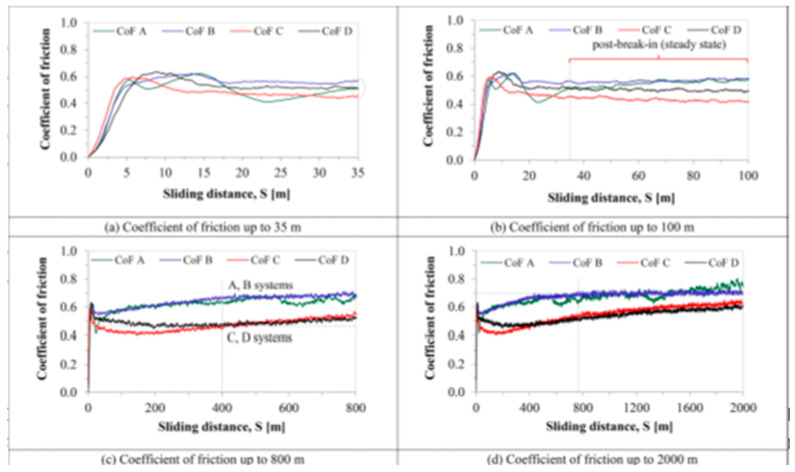
Change in the CoF with sliding distance. (**a**) AISI H13 steel; (**b**) H13 + SM-SEDM]; (**c**) H13 + SM-SEDM + TiAlN; (**d**) H13 + SM-SEDM + SR + TiAlN [90]. (With kind permission from Elsevier).

The tribological properties of a material, such as the coefficient of friction (CoF), wear resistance, etc., are studied using various kinds of tribometers; the pin-on-disc tribometer is the most common of them all. A pin is situated perpendicular to the disc-shaped workpiece, which is pressed against the circling disc by a tester with a fixed force and speed. da Silva et al. [90] investigated the effect of coating and die-sinking electrical discharge machining on the surface of annealed AISI H13 steel. The objective was to observe how the co-efficient of friction and wear resistance changed on the EDMed surface, which was modified by the electro-discharge coating process; a MICROTEST MT/60/NI tribometer was used to measure those properties. Urea was mixed with deionized water dielectric fluid, and a copper tool electrode with straight polarity was used to modify the machined surface. Tungsten carbide was used as the pin material on the tribotester. The authors examined the friction behavior of the workpieces and measured the co-efficient of friction (CoF) at a sliding distance of up to 2000 m (shown in Figure 11). The values of CoF became steady when all workpieces experienced a sliding distance of more than 35 m. This was due to the balance among the factors, which affected the friction, therefore achieving smoother surfaces. The CoF tended to increase after the sliding distance reached 400 m in the case of coated workpieces. The presence of an oxide layer on the surface of the coated workpiece resulted in a lower CoF as the EDM-coated layer acted as lubricant. On the other hand, annealed- and EMDed-only surfaces had higher CoF values due to stick slipping and the existence of debris, respectively. Ferrous (Fe) and chromium (Cr) were found on the pin’s surface, and tungsten was found on the worn track, which indicated the increase in the CoF for the coated workpiece after 400 m sliding distances. The worn track of the annealed steel workpiece ensured the plastic deformation as the pressure applied by the pin was greater than the shear strength of the material. This plastic deformation resulted in a higher material loss of the annealed workpiece. Abrasive wear on the track was found on the EDMed surface, as well as grooves and scars. The wear tracks were shallower and smoother in case of the coated workpiece. It was also found that the surface wear rate (SWR) decreased with the increase in hardness. As the coated surface possessed the highest hardness and higher load carrying capacity, it experienced the lowest SWR. The SWR in the case of the annealed workpiece was 4–6 times that of the coated workpiece due to its lowest hardness.

Delgado et al. [91] used wire-EDM to machine WC-Co, WC-Ni, and ZrO_2_-based composites and studied the tribological performance of the machined surface by studying the effect of the load and surface finish on the CoF values and wear characteristics of the machined surfaces. A pin-on-flat tribometer was used for analyzing the tribological characteristics of the EDMed surface. The static and dynamic CoF values were found within the range of 0.52–0.82 and 0.32–0.52, respectively.

**Figure 12:**
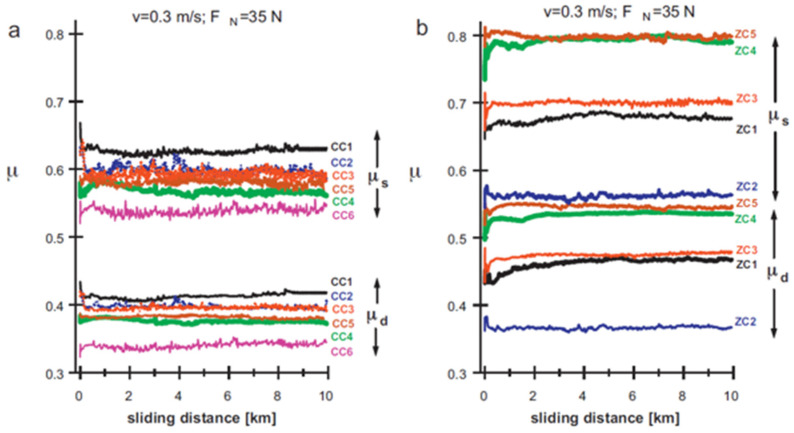
Change in the coefficient of friction (CoF) with the sliding distance for different samples of (**a**) cemented carbides and (**b**) ZrO_2_ based composites [91]. (With kind permission from Elsevier).

The friction curves tended to increase at first, then declined and finally increased again in order to achieve an equilibrium state (shown in Figure 12). They also observed that the friction curves fluctuated in the whole wear experiment. The micro-junctions that were situated on the contact surface experienced continuous breaking and regeneration. As a result, the interaction between the contact surface and the pin changed with the sliding distance. The tribological properties also highly depended on the surface finish. The CoF was the lowest in case of WC-Ni grade CC6. Moreover, the CoF as well as the wear level were the highest in the case of ZrO_2_-TiN grade ZC5.

Philip et al. [92] used a Cu electrode in the EDM process to modify the surface of Ti-6Al-4V and investigated the tribological properties by using a pin-on-disc tribometer at several high temperatures (200 °C, 400 °C, and 600 °C) and by varying an applied load ranging from 50 to 150 N. The hardness of the EDMed workpiece increased four-fold due to the formation of several oxides such as TiO_2_, Ti_8_O_15_, and Ti_24_C_15_. At 200 °C and 100 N, the tribological performance was much improved because of the formation of oxide layers; however, at higher temperatures and applied load (150 N), the materials were worn out by spalling. It was also found that the formation of Ti_24_C_15_ not only resulted in a high hardness but also protected other oxides and played a noticeable role against wear.

### 4.2. Surface Characterization and Composition Analysis by SEM and EDS

Scanning electron microscopy (SEM) and energy dispersive spectroscopy (EDS) are used worldwide by numerous researchers for surface characterization and composition analyses. The white layer or recast layer is a common phenomenon when an electrically conductive material undergoes EDM processing. EDM involves instant melting and vaporization of a workpiece at a very high temperature (~10,000 °C). The dielectric fluid flushes away the debris produced in EDM. Some of the melted particles do not get flushed and resolidify over the EDMed surface during the pulse off-time, thus creating a layer known as the white layer. The microstructure highly depends on the properties of the electrode and dielectric fluid used as well as the process parameters. Beyond the heat affected zone (HAZ), the microstructure of the parent material does not change. The existence of the white layer can be identified in SEM micrographs, and the elements/compounds that make up this layer can be recognized by EDS analyses.

**Figure 13:**
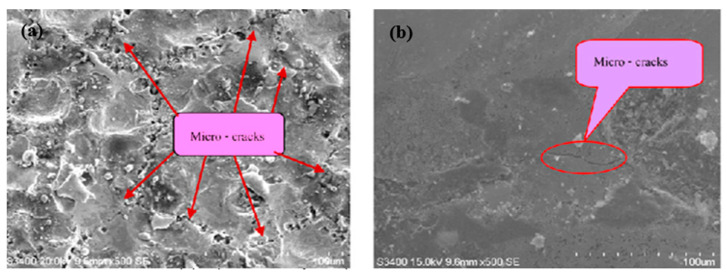
SEM images that represent the surfaces of tungsten carbide produced by (**a**) EDM and (**b**) EDC [87]. (With kind permission from Elsevier).

SEM analysis generates electron beams that strike and produce signals that contain information of the surface [93,94]. These informative signals are then converted to images. EDM process parameters such as peak current, pulse on-time, pulse off-time, etc., play a vital role in modifying the machined surface. Researchers use SEM in order to analyze the surface characteristics, which include the recast layer thickness, voids, cracks, globules, pockmarks, etc. of the EDMed surface. An AA606/10% SiC composite surface was modified by PMEDM [95]. The accretion of molten materials from the workpiece, tool electrode, and dielectric along with the powder particles developed a homogenous recast layer. The added tungsten powder resulted in a lower impact force on the surface and decreased the energy density as well, thus resulting in a smoother surface. Janmanee and Muttamara [87] compared the surfaces of tungsten carbide prepared by electro-discharge machining (EDM) and electro-discharge coating (EDC) processes using titanium powder suspension. The SEM micrographs, which are shown in Figure 13, confirmed that the EDC process produced a flatter recast layer with less microcracks compared with the EDM process when the current and duty factor were kept constant. The titanium powder entered into the microcracks and voids, resulting in a smoother surface and lower crest height in the case of EDC. This was also the reason behind the lower roughness of the surfaces prepared by EDC. The effect of current was studied, and the authors found that at current = 20 A and duty factor = 50%, the optimum Ti coating was 20.32%, revealing that this current was suitable for the experiments. The bonding ability decreased with the increase in current from 20 A. They also studied the effect of the duty factor and found that at duty factor = 50%, the optimum Ti coating was 21.46% and the thickness of that coating was 5 µm.

Bhaumik and Maity [96] used different tool electrodes to machine Ti alloy and investigate the effect of these tool electrodes on recast layer thickness and surface topography. The SEM images showed that the smoothest and thinnest recast layer was obtained when a copper electrode was used compared with brass and zinc electrodes, whereas the surface with the highest defects and non-uniformity was achieved by using a zinc electrode. It was found that the recast layer thickness increased with the rise in peak current during the EDM process. Dewangan et al. [97] investigated the white layer thickness and found that the white layer thickness was the lowest when brass was used as an electrode material followed by copper and graphite at peak current 8 A. The lowest thermal conductivity of graphite among the electrodes used resulted in the lowest heat dissipation during EDM. Moreover, the white layer thickness was directly proportional to the peak current and pulse on-time because of the higher discharge energy effect that was identified in the SEM micrographs.

Energy dispersive spectroscopy (EDS) is an analysis that is widely used to find the percentage composition of the machined surface. An electron beam is used in this process, which is focused on the EDMed surface. The incident beam of the electron hits the surface, which excites the electron within the atom of the EDM sample. The electron ejects from its shell, thus creating a hole in the lower energy shell of that atom. To fill the gap, an electron from a higher energy shell jumps to the lower energy shell. The energy that is released in the form of an X-ray due to the ejection of an electron from a lower energy shell and the filling of that hole by another electron from a higher energy shell can be measured using an energy dispersive spectrometer. The X-ray energy found in this process is different for different elements, so this analysis provides the composition of the elements found on the machined surface [98]. The migration of elements from the dielectric fluid and electrode as well as the chemical composition of the white layer can be identified by EDS analysis.

Bhaumik and Maity [96] analyzed the chemical composition of the white layer that was formed after machining the titanium alloy surface with three different electrodes (i.e., copper, zinc, and brass electrodes). The EDS analysis confirmed the existence of electrode material present on the machined workpiece surface. The high temperature associated with the EDM process resulted in the decomposition of dielectric fluid, and thus the carbon migrated from the dielectric to the machined surface.

**Figure 14:**
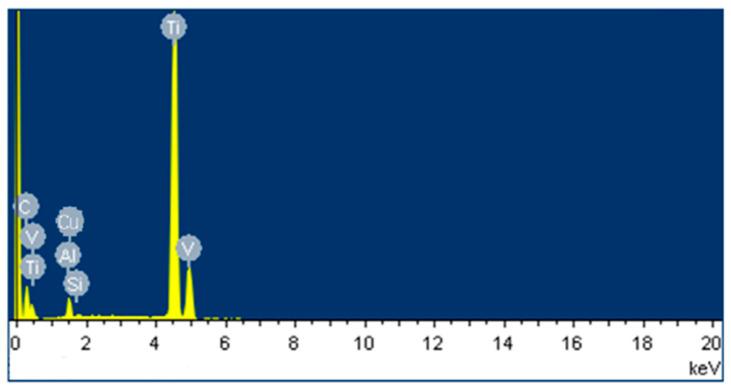
EDS analysis of machined Ti-6Al-4V alloy when Cu-SiC electrode was used at current = 1.5 A, pulse on-time = 15 µs, and pulse off-time = 15 µs [68]. (With kind permission from Elsevier).

Li et al. [68] used a conventional Cu electrode and Cu-SiC composite electrode to machine the Ti-6Al-4V alloy by the EDM process. The EDS analysis (Figure 14) showed that the carbon content was higher on the machined surface because of the EDM oil used in this process; the authors also found the presence of Si and Cu on the machined surface. The surfaces produced by both electrodes produced a harder layer than the base material. Sharma et al. [59] modified the surface of biodegradable Mg/Zn alloy by using a powder-mixed EDM process.

The EDS analysis ensured that the powder deposition rate onto the machined surface increased with the increase in powder concentration. This deposition rate was higher in the case of zirconium powder than in manganese powder. Manganese has a lower melting point and the EDM process generates a very high temperature in the sparking gap. Therefore, manganese powder partially vaporized in this process, resulting in a lower deposition rate.

### 4.3. Phase Characterization Using the XRD Technique

**Figure 15:**
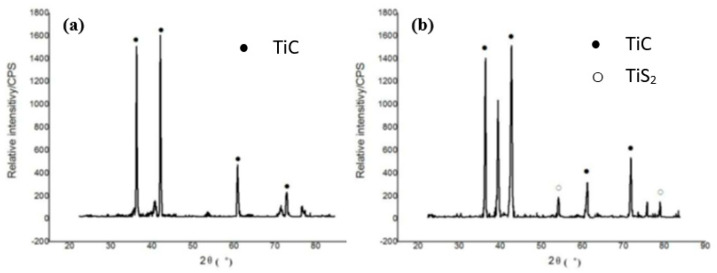
XRD patterns when EDM was conducted on Ti-6Al-4V alloy: (**a**) using Cu electrode at current = 1.5 A, pulse on-time= 15 µs, and pulse off-time = 15 µs; (**b**) using Cu-SiC electrode at current = 1.5 A, pulse on-time = 15 µs, and pulse off-time = 15 µs [68]. (With kind permission from Elsevier).

X-ray diffraction (XRD) is another analysis performed to evaluate the molecular structure of the crystals formed in the EDM process. A beam of X-rays is incident onto the workpiece. The crystals diffract those X-rays in different directions. The angles and intensities of the X-rays were measured to find the electron density as well as their chemical bonds [99]. This analysis plays an important role in understanding the micro-hardness of the workpiece before and after it undergoes the EDM process. Micro-hardness primarily depends on the percentage of carbon present in a sample. The more the percentage of carbon exists in a sample, the more micro-hardness is expected. Li et al. [68] performed an XRD analysis after separately machining Ti-6Al-4V alloy with a conventional Cu and Cu-SiC electrode, which is shown in Figure 15. The XRD analysis displayed that TiC was produced by the chemical reaction between the work piece and the carbon found from the dielectric oil. TiS_2_ was produced due to the chemical reaction between the workpiece and SiC. The surfaces formed by using CuSiC electrode showed a higher hardness than the surfaces produced by the conventional Cu electrode. This is because of the presence of TiC and TiS_2_ on the machined surface.

Batish et al. [100] used EN31, H11, and high-carbon high-chromium (HCHCr) as workpieces to investigate the effect of micro-hardness using different electrodes and dielectrics. EN31 was machined by using copper electrode and aluminum powder was mixed with dielectric kerosene oil. The XRD analysis traced the presence of carbon, aluminum, and copper onto the machined workpiece surface. An amorphous region was formed due to the rapid cooling of the workpiece. This region increased the overall hardness as well as the brittleness of the machined EN31 material. An XRD analysis was also performed on the EN31 material that was machined using a tungsten-copper electrode and aluminum-mixed dielectric. The presence of Al-C onto the machined surface ensured a better surface finish and higher hardness. An amorphous structure was also visible from the XRD analysis performed on the H11 material machined by the copper electrode and kerosene mixed with graphite powder as the dielectric. The reason behind the formation of this amorphous structure was the higher pulse on-time, lower pulse off-time, and rapid cooling of the workpiece. Fe_3_C was formed due to the EDM, which plays an important role in increasing the hardness. Moreover, the copper percentage also increased after the machining, which was detected by the XRD analysis. The same workpiece was machined using a different powder (aluminum particles). The XRD analysis traced the existence of aluminum on the machined surface, which entered into the craters, thus decreasing the surface roughness. Cohenite was formed, which was responsible for increasing the hardness. Additionally, the copper percentage increased as before. They also performed the XRD analysis on the HCHCr material, which was machined using tungsten-carbide electrode and refined mineral oil mixed with graphite powder as the dielectric. Chromium iron carbide and iron carbide were formed due to the high temperature involved in the EDM process. The iron in the chromium iron carbide came from the cementite phase, which increased the hardness and reduced the overall mechanical stress. Moreover, the carbon percentage was increased on the machined surface. The same material was machined by a copper electrode and kerosene dielectric mixed with graphite powder. The XRD analysis found the existence of hepta-chromium-tri-carbide (Cr_7_C_3_) on the EDMed surface. Cr_7_C_3_ possesses a very high hardness, which could be the potential reason for the increased surface microhardness of the EDMed surface. Crystalline structures were mainly formed due to the higher pulse off-time.

## 5. Application of Electro-Discharge Surface Modification and Coating

### 5.1. Surface Modification for Protective Coating

**Figure 16:**
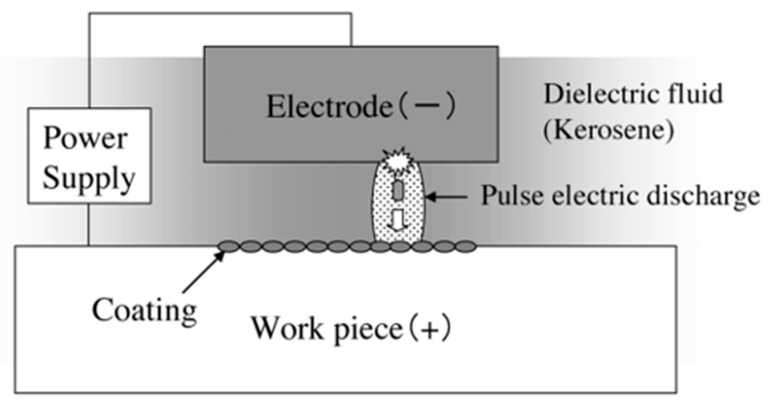
Schematic diagram representing the mechanism of the EDC process [112]. (With kind permission from Elsevier).

Different types of surface modification methods, such as electroplating [101,102], carburizing [103], oxidation [104,105], ion implantation [106], chemical vapor deposition [107], laser treatment [108,109,110,111], etc. are nowadays being used to modify surfaces. The majority of the mentioned manufacturing processes require multiple steps or techniques. On the other hand, EDM can provide a simple solution to this problem by simultaneously performing both the coating and machining processes. The EDM process can be used to modify the surfaces of hard-to-cut materials and/or create protective surface coating for various functional applications. This non-conventional process has tremendous potential and can be used for machining complicated parts for automotive, aerospace, and biomedical sectors. As discussed in an earlier section, the materials and alloys need to be modified or coated to survive extreme working conditions. A protective layer can also be formed on the workpiece by machining the surface using the electro-discharge coating (EDC) process. The main difference between EDM and EDC is the connection of electrodes to the power supply. In EDC process, the tool and workpiece electrodes are connected to the anode and cathode, respectively. Figure 16 shows the schematic diagram of the EDC process. Many research works have been conducted to investigate the protective layer formed during the EDC process by varying the process parameters and/or by the inclusion of target materials in the form of powder metallurgy electrode or additives in the dielectric.

Chen et al. [113] used a dry EDC process to produce a TiN film over the surface of Al 6061 alloy. The material deposition rate (MDR) increased with the increase in current and pulse on-time. The EDX analysis confirmed a homogenous distribution of Ti/Al/N atoms over the parent material. TiN along with Ti and N_2_ atoms strongly adhered with the base material when the peak current was increased. This is because of the higher discharge energy produced during the higher peak current, which resulted in a higher heat energy production in the process. However, a rougher TiN film was produced at a higher peak current along with TiN and AlN films. It was concluded from the study that the optimal process parameters should be adopted for the uniform deposition of TiN film on the machined surface to avoid a significant number of micro-cracks. Patowari et al. [66] developed a thick protective layer of W_2_C over C-40 steel using the EDC process. The existence of C at the deposited layer played a vital role in increasing the microhardness.

### 5.2. Surface Modification for Enhancing Biocompatibility of Implants

With aging, a lot of people suffer from different kinds of inflammatory diseases, which include rheumatoid arthritis, osteoporosis, and chondromalacia. These diseases result in bone decay when the bones experience cyclic loading and eventually suffer from complete functional loss [114]. These bones or joints can be replaced by introducing implants into the body system [115]. In recent years, it has been reported that the EDM process has the potential to modify implant material surface in a favorable condition for improved biocompatibility. The modified surface after machining by EDM was found to generate a protective oxide layer with micro- and nano-scale porosity that would improve the corrosion resistance and biocompatibility of the implant material [43]. Several studies have been carried out investigating different strategies of improving the biocompatibility of the implant surface using EDM. Sharma et al. [59] performed a corrosion performance test on an EDMed Mg/Zn alloy to investigate its biofunctionality. The analysis was conducted using simulated body fluid (SBF) as the corrosive environment for 28 days (672 h). The volume of SBF was 380 mL and 370 mL in the polished sample and machined sample, respectively. The SBF ion concentration was balanced in such a way that it was close to that of human body fluid. The polished sample consisted of larger pits as well as cracks and resulted in a higher corrosion rate because the pits and cracks activated the galvanic cell mechanism on the alloy surface. On the other hand, a carbonaceous layer of Zr was developed on the alloyed surface in the case of the EDMed sample, which acted as a barrier against corrosion. The SBF could not come in contact with the machined surface due to the layer. As a result, the Mg alloy transfer rate from the surface was lower. A lower corrosion rate is expected in the biomedical applications, as the alloy should stay in an integrated form until the fracture bone is fully recovered. The results showed that zirconium powder was better than magnesium powder when used in PMEDM to increase the biofunctionality and corrosion resistance of Mg/Zn alloy.

It was found that the surface roughness ranging 1–2 µm is allowable for oral implants [116]. Hsieh et al. [117] used die-sinking EDM to investigate the surface roughness as well as biocompatibility of Ti_50_Ni_50_, Ti_50_Ni_49.5_Mo_0.5_, and Ti30Nb1Fe1Hf using distilled water as the dielectric fluid and titanium as the tool electrode. The thickness of the generated recast layer, which was formed due to the machining, was non-homogenous. The defects formed on the recast layer were trivial, and therefore, this layer was firmly attached to the TiNi and TiNb surfaces. A TiO layer was found on the TNM and TNB surfaces. Moreover, Nb and Ni oxides formed on the Ti30Nb1Fe1Hf and Ti_50_Ni_49.5_Mo_0.5_ surfaces, respectively. The surface roughness was the highest on the TN, followed by the TNM and TNB surfaces. However, each of the samples exhibited a surface roughness ranging from 1–2 µm when current was 1–2 A and pulse duration was 10–20 µs. Therefore, the machined surfaces of these alloys after EDM are suitable as oral implants surfaces under lower pulse on-time and current conditions. TiO_2_ and WO_2_ layers were generated on a Ti-6Al-4V workpiece surface after micro-EDM using a tungsten carbide tool, as reported by Jahan et al. [118].

The surface roughness as well as the crater sizes decreased with the decrease in applied voltage and discharge energy. The surface roughness was less than 100 microns, which is suitable in terms of cell adhesion and proliferation [119].

**Figure 17:**
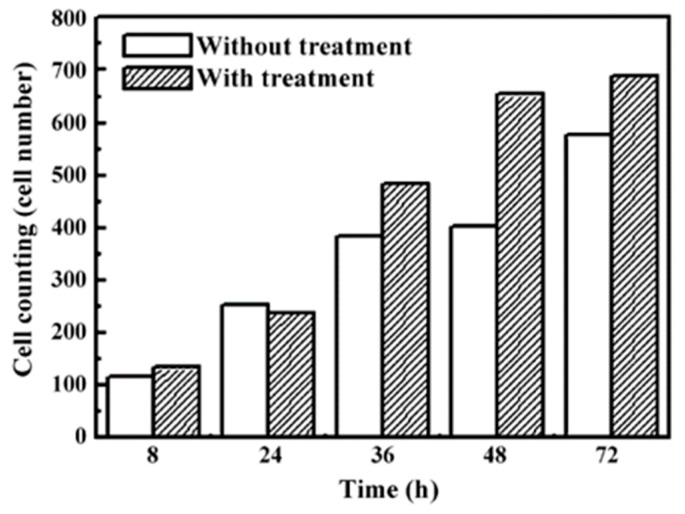
Cell counting performed on the treated and EDMed Fe-Al-Mn alloy [122]. (With kind permission from Elsevier).

**Figure 18:**
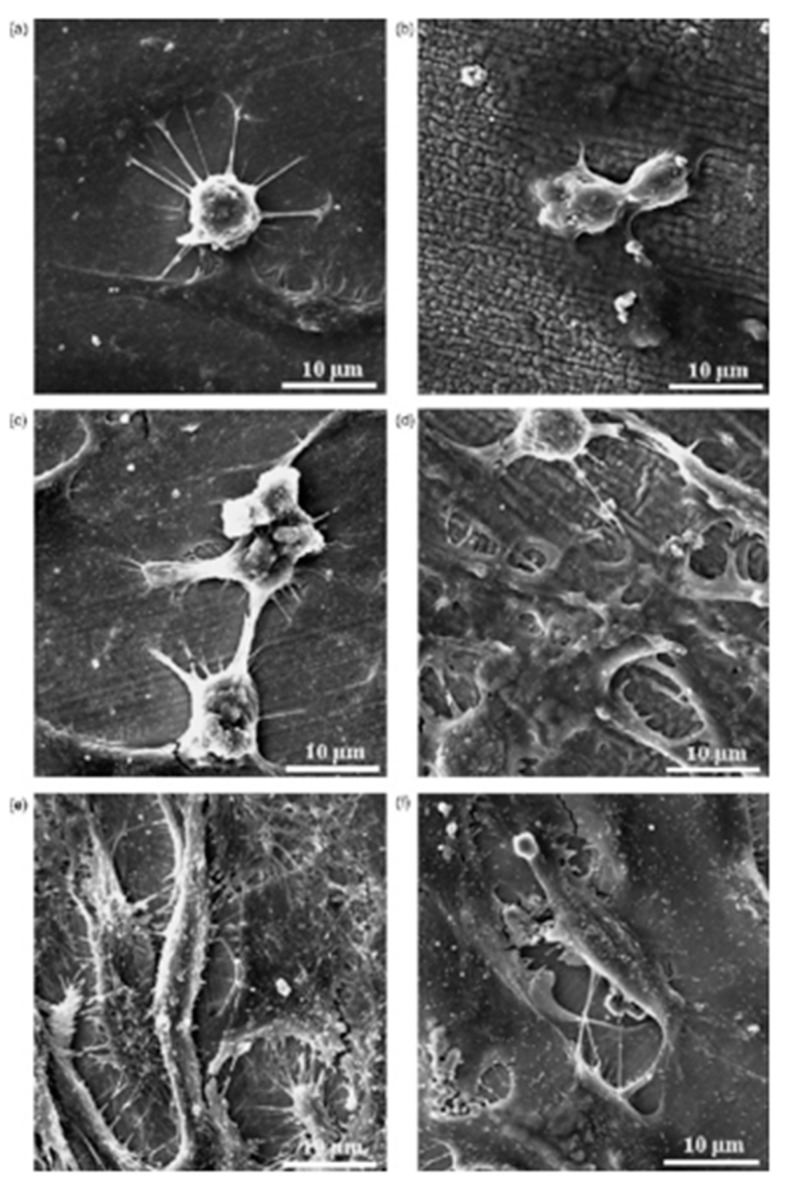
SEM images of the untreated and EDMed Fe-Al-Mn alloys at different culture times: (**a**,**b**) 8 h, (**c**,**d**) 24 h, and (**e**,**f**) 48 h [122]. (With kind permission from Elsevier).

Fe-Al-Mn alloys are widely used in dental and orthopedic implants due to the oxide layer formed on these alloys. An oxide layer on the dental surface can protect the implant inside the human body system, and it is quickly recovered in the case of any damage [120]. However, modified implants are required nowadays to speed up the recovery procedure [121]. Chen et al. [122] compared the biofunctionality of untreated and EDM-treated Fe-Al-Mn alloy. These alloys were prepared in an air induction furnace, whereas the EDM was used for the treatment of Fe-Al-Mn alloy using a copper electrode. A micro-scale recast layer was generated on the implant material, and the thickness of this newly formed recast layer depended on the field-assisted migration of ions from the dielectric medium (kerosene). The nano-porosity and thick oxide layer formed on the machined surface improved the implant’s performance in terms of cell adhesion and corrosion resistance. The cell morphology (shown in Figure 17) and the SEM micrographs (shown in Figure 18) confirmed that the cells spread in both the treated and untreated alloy. The cells were found to be round with protruding nuclei in both alloys after 8 h of cell culturing. The cells were surrounded by cytoplasm (Figure 18b). After 24 h, the cells were flat, well-spread, polygonal, and had protruded nuclei in the untreated alloy, whereas cells that had no protruding nuclei and unclear orientation were observed in the treated alloy.

The cells became more elongated as time passed. After 48 h, significant growth of cells, irregularities, and strong adhesion to the surface were observed. The cells on the treated alloy surface had better cell adhesion. The reason behind higher cell growth in treated alloy might be due to the larger area of the surface interacting with cells compared with the untreated alloy. No significant distribution of cells was observed until after 4 h; significant cell distribution was observed after 8 h. As time passed, the cell attachment percentage increased in the case of the treated alloy. It was concluded that the EDM process in this alloy resulted in an improved biocompatibility and osseointegration.

**Figure 19:**
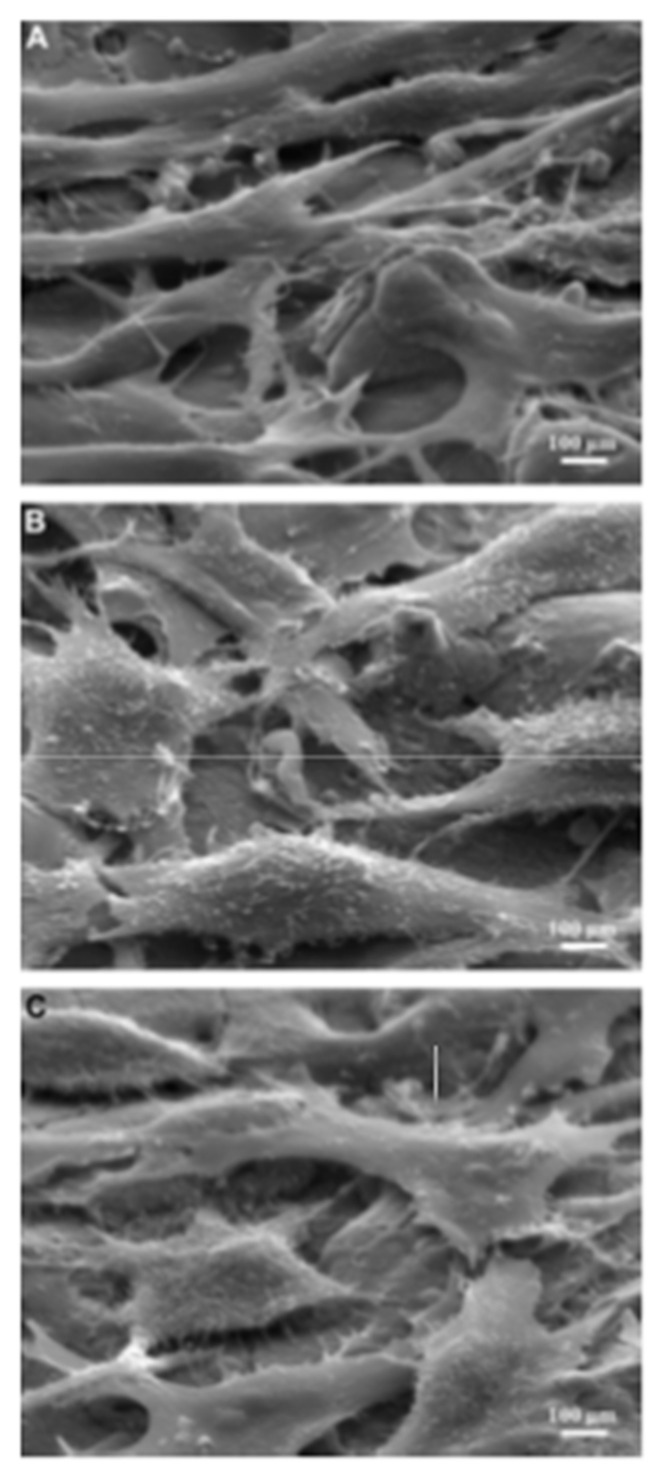
MG-63 cells attachment and proliferation after 7 days of cell culturing on the (**A**) untreated Ti64 specimen, (**B**) EDMed Ti64 specimen at 10 s pulse on-time, and (**C**) EDMed Ti64 specimen at 60 s pulse on-time [123]. (With kind permission from Elsevier).

Lee et al. [123] investigated the surface modification of Ti64 alloy for cell culture performance with the osteoblastic cell (MG-63). Ti was used as the tool electrode, and the pulse duration was varied within a range of 10–60 µs. The untreated specimen showed a relatively finer surface whereas globules and pits were found on the EDMed surfaces. The surface roughness increased with the increase in pulse duration. The adhesion and proliferation of MG-63 cells are shown in Figure 19. The MG-63 cells were adhered to both the untreated and treated specimens. However, the osteoblastic cells spread entirely on the EDMed specimen. Pores of the treated specimens were penetrated by filopodia. The density of the cells was higher on treated specimen with the increase in culture period. It was found that the EDM process not only affected the surface roughness but also affected the wettability of the treated surface. The EDMed surface exhibited hydrophilicity, whereas a hydrophobic property was displayed by the untreated specimen. It was suggested that the processed Ti64 alloy by EDM can be widely used in clinical purposes due to its significant adherence and proliferation of MG-63 cells without any side effects.

Jahan et al. [30] used micro-EDM to modify the surfaces of Ti-6Al-4V and NiTi alloys to explore the biocompatibility of the modified surfaces. The formation of debris was higher in the case of the Ti-6Al-4V alloy surface compared with the NiTi alloy surface, which resulted in a smoother surface of NiTi compared with Ti-6Al-4V. The surface roughness was different at the edge and center due to the variation of sparks. TiO_2_ and WO_2_ layers were formed in the case of the Ti-6Al-4V alloy, and a NiTiO_3_ film was formed over the NiTi surface after these workpieces were machined by micro-EDM. These oxide layers prohibited the release of ions from the base material and thus acted as a barrier. As a result, the biocompatibility of the alloy can be notably increased. In another study, it was found that tool wear can be significantly decreased at a higher rpm using coated tool electrodes compared with uncoated tools by adopting micro-EDM processing [124].

The surface of a Ti-6Al-4V alloy was modified by a combination of EDM, acid etching, and shot peening in the study by Strasky et al. [125]. The surface was etched using hydrofluoric acid (HF), nitric acid (HNO_3_), and sulfuric acid (H_2_SO_4_). MG-63 cells were further cultivated for 7 days in order to look into the cell proliferation and biocompatibility of the surface. The white layer along with the heat affected zone was formed due to EDM, which was removed by etching. Etching and etching followed by shot peening changed the bioactivity of the EDMed surface. Shot peening drastically lowered the surface roughness. It was found that etching played a significant role on the cell growth over the surface as the surface became more wettable in this process. According to the SEM images, the surface topography of the EDM + etching and EDM + etching + shot peening surface were almost identical. However, the latter resulted in a higher DNA concentration. It was assumed that the variation of the chemical composition of the two surfaces was the reason behind the difference in DNA concentration. It was concluded that the three-step process was a better strategy in order to manufacture implant materials.

**Figure 20:**
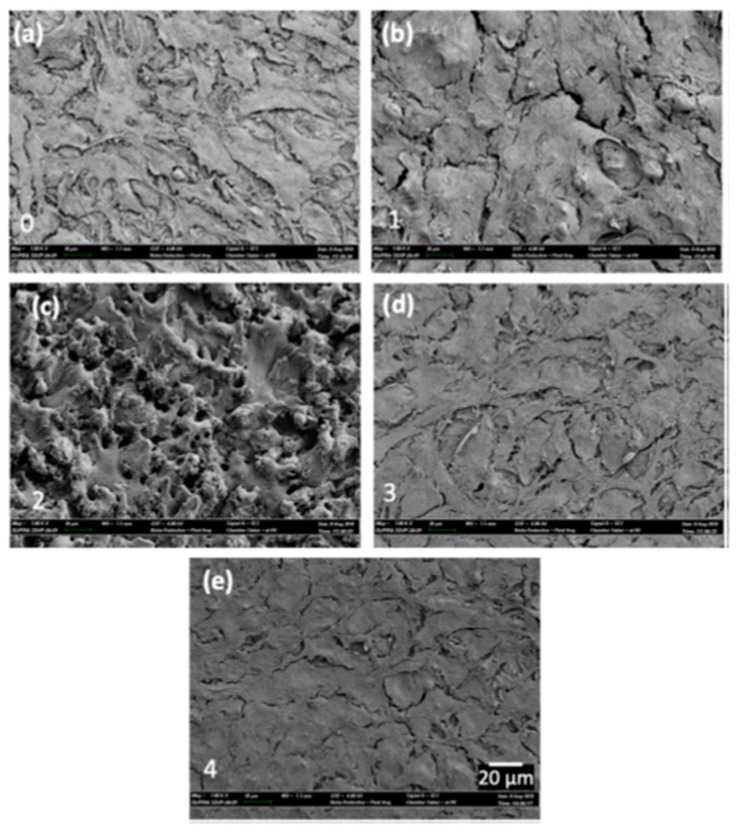
SEM micrographs showing the attachment of MG-63 cells on (**a**) conventionally machined surface and (**b**–**e**) samples prepared by WEDM by different parameters [43]. (With kind permission from Elsevier).

Mahbub et al. [43] modified the surface of grade 5 titanium alloy by the wire-EDM (WEDM) process in order to investigate the effects of surface and sub-surface characteristics on the biocompatibility and corrosion performance. The cell attachment and cell proliferation response of mouse osteoblastic cell MC3T3-E1 were studied to determine the biocompatibility. The presence of these phases helped in cell attachment, which are shown in Figure 20. The smoothest surface (Figure 20e) exhibited the highest cell attachment because of the highest percentage of β and α + β phases at the subsurface. The corrosion rate on various samples were also examined, and it was found that the corrosion rate decreased with the decrease in surface roughness. The lowest corrosion rate of 12.13 mils/year was found in the case of the smoothest surface, whereas the maximum corrosion rate of 21.04 mils/year was found in the case of the roughest sample. A titanium oxide layer formed due to the EDM process, which prevented the implant material from being corroded, thus improving the biocompatibility.

Chakmakchi et al. [126] modified the surface of Co-Cr and Ti dental alloys by the EDM process and conducted an inquiry into the modified surface for corrosion performance. It was established that the use of a Cu electrode had a deleterious effect on the corrosion resistance of Ti alloy. On the other hand, when a Ti electrode was used, the change in the electrochemical properties was negligible compared with the Cu electrode; therefore, a Ti electrode is a better option to use when implants are created by the EDM process. Al-Amin et al. [127] modified the surface of 316L steel. This alloy is widely used in manufacturing biomedical devices, although several failures of these devices have been observed because of fatigue, corrosion, and wear [128]. Hydroxyapatite powder (HAp) was mixed with the dielectric, and titanium was used as the tool electrode. A uniform and thin coating of 15.29 µm was formed onto the surface after the EDM processing. Furthermore, a few craters and micro-cracks were observed onto the modified surface. Several analyses found the existence of CaTiO_3_, CaCO_3_, Ti, HA, and Ca_4_(PO_4_)_2_O on the EDMed surface. Moreover, the presence of TiC and Cr_3_C_2_ enhanced the surface microhardness by 80%. Over 70% of living cells alongside a couple of dead cells were obtained for the modified, hydrophilic 316L steel. This guaranteed an improved cell viability, proliferation, and biocompatibility.

Davis et al. [129] modified the surface of Mg Alloy AZ91D by powder-mixed micro-EDM. The concentration of the powder was varied in the range of 0–8 g/L. The recast layer was glossy, and the workpiece gained hydrophobicity, which resulted in less corrosion. Therefore, this modified alloy can be used in biomedical sectors. Furthermore the authors modified the surface of medical-grade Ni_55.6_Ti_44.4_ alloy by micro-EDM processing using a Zn powder particle [63]. Cu and brass electrodes were used to compare the effect of different powders on the modified surface. Additionally, the concentration of the powder particles was varied. The copper electrode resulted in a better machining time, dimensional accuracy, and higher recast layer thickness compared with the brass electrode. Moreover, a noticeable modification on the recast layer was observed, which resulted in an enhanced microhardness; however, the surface roughness also increased. The modified workpiece displayed noteworthy noncytotoxic behavior towards the cell. Additionally, the higher cell viability percentage ensured a tremendous role of the modified alloy in the broken tissue recovery. The results found by cytocompatibility and wettability, as well as topographic and microhardness tests revealed that this modified alloy is suitable to use in implants for cardiovascular applications. Recent works on this sector using EDM are summarized in Table 2.

### 5.3. Surface Modification for Improved Tribological Performance

**Figure 21:**
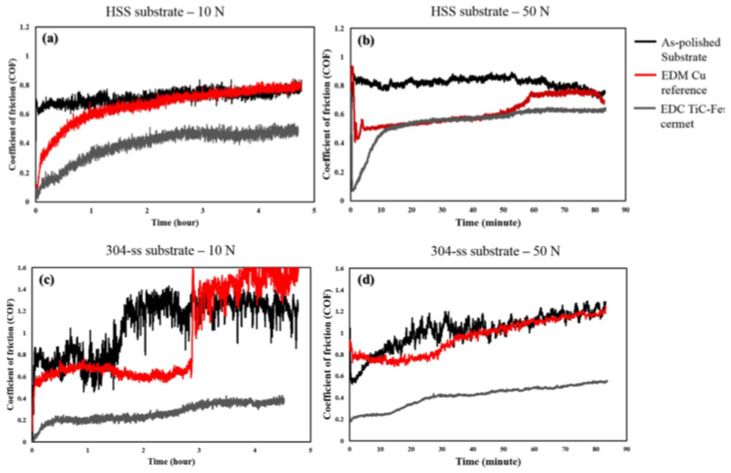
A comparison of the time-dependent coefficient of friction of the as-polished substrate and the coated and EDMed samples at various loads: (**a**,**b**) HSS substrate and (**c**,**d**) 304 SS substrate [130]. (With kind permission from Elsevier).

Several research studies have been conducted by varying the workpiece material and EDM conditions to examine the tribological performances, such as the wear resistance and coefficient of friction of a material or alloy. Algodi et al. [130] used a semi-sintered TiC tool electrode to modify the surface of high-speed steel (HSS) and 304 stainless steel by the electrical discharge coating (EDC) method. The average surface roughness was slightly greater in the case of EDM-coated 304 stainless steel compared with HSS. The wear resistance was performed by adopting a dry sliding reciprocating technique. Two conditions, such as 10 N and 35,000 reciprocating cycles and 50 N and 10,000 reciprocating cycles, were considered. The wear rate was found lower in the case of the coated HSS and stainless steel than the as-polished HSS and stainless steel in both loadings, respectively. The wear resistance increased in both coated specimens due to the increased hardness as a result of the layer that formed on the workpiece surfaces. Figure 21 shows the comparison of the coefficient of friction (COF) for each sample. The COF decreased in both coated specimens compared with the as-polished samples and surfaces machined by EDM processing using a Cu electrode. The coated stainless steel resulted in a higher value of COF (~0.6) compared with the HSS (~0.2) at a steady-state condition. The coated stainless steel experienced less wear resistance and a lower COF value than HSS due to the lower hardness of the stainless steel. However, the overall tribological performance of both coated steel workpieces increased compared with the as-polished substrates.

**Figure 22:**
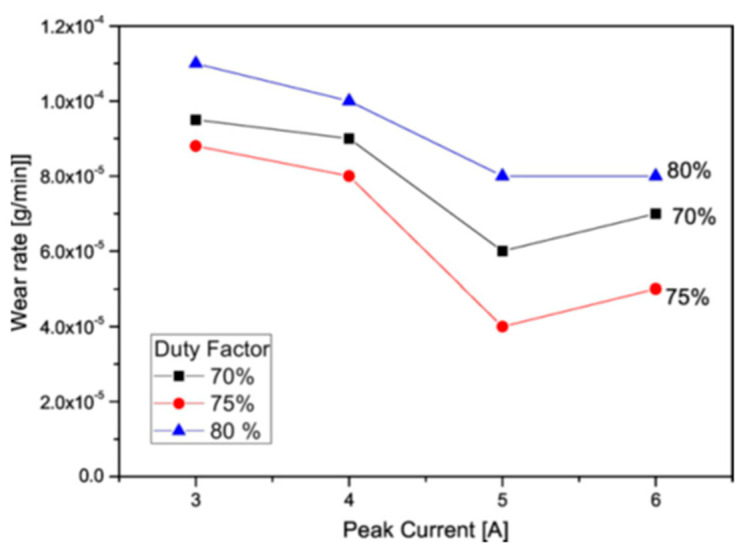
Change in wear rate with respect to the peak current and duty factor when the TiC-TiB_2_-coated Ti alloy experienced the ball-on-disc sliding wear test [85]. (With kind permission from Elsevier).

In a similar study by Tijo and Masanta [85], Ti-6Al-4V alloy was modified by EDC using Ti and B_4_C powder in a kerosene dielectric fluid. A composite coating of TiC-TiB_2_ was formed on the alloy surface in this process. As a result, the hardness of the coated alloy notably increased compared with the uncoated alloy. A WC-Co ball was used against the workpieces to conduct a sliding wear test. The coated alloy showed a 2–7-fold higher wear resistance compared with the uncoated alloy. The lower wear rate of the coated surface was associated with hard phases on the coated surface. The effect of the peak current and duty factor on the wear rate is shown in Figure 22. The wear rate decreased with the increase in current at first, then increased with the further increase in current. Moreover, the wear rate followed a similar trend in the case of duty factor. A lower concentration of the hard TiC-TiB_2_ phase increased the wear rate. A layer produced at 80% duty factor contained a significant number of pores on the surface.

As a result, the removal of coating was accelerated at an 80% duty factor. The result of the wear track showed that the Ti alloy without coating had a higher wear track depth (~55 µm) compared with the EDM-coated Ti alloy (14–20 µm).

A Ti-6Al-4V alloy was machined by Sharma et al. [45] using micro-EDM, where hexagonal boron nitride powder was mixed with the deionized water dielectric. The pin-on-disc test was conducted to find out the tribological performance of the EDM-coated and uncoated alloy surfaces. The wear rate and coefficient of friction was lower in the case of the coated alloy surface compared with the uncoated Ti alloy. Slipping occurred between the two adjacent coated layers and therefore, the coefficient of friction decreased. It was also found that the wear rate decreased with the increase in the duty factor because of the higher hardness of the coated layer. The maximum wear rate of 0.22 mg/min was found on the base material, and the minimum wear rate was 0.05 mg/min, which was found on the coated Ti alloy. The coefficient of friction decreased with the decrease in duty factor. Wear grooves that were parallel to pin sliding ensured an abrasive wear-type mechanism. This was because of the existence of grooves on the coated surface whose hardness was higher than the parent unmachined material.

**Figure 23:**
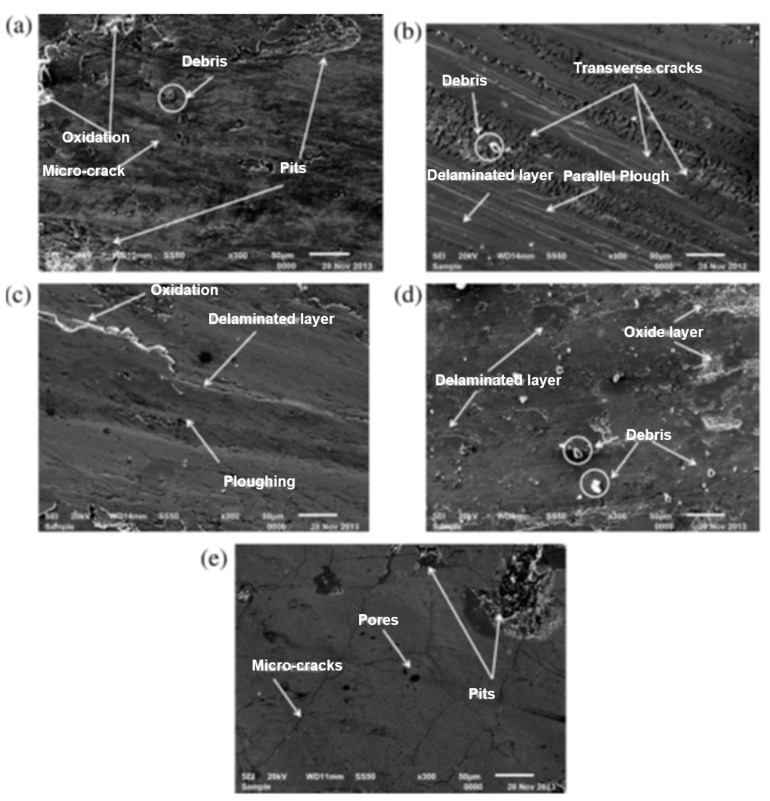
Coated alloy after wear at (**a**) room temperature, (**b**) 100 °C, (**c**) 200 °C, (**d**) 300 °C, and (**e**) 400 °C [131]. (With kind permission from Elsevier).

Arun et al. [131] successfully deposited nickel and tungsten onto the AISI-D2 die steel surface by an electrical discharge alloying (EDA) process. A pin-on-disc tribometer was used to examine the tribological behavior of the alloyed surface. The specific wear rate increased with the elevation in temperature, which can be seen from the SEM images in Figure 23. Trivial amounts of debris were formed at room temperature due to the high hardness of the carbon content.

On the other hand, the oxide layer that was formed at the high temperature was negligible. As a result, more elements such as, Ni, C, W, and WC migrated to the contact layer, and the specific wear rate was increased. This was also the reason behind the lower coefficient of friction at higher temperature, as these elements played the role of the lubricant at the contact surface. Moreover, the peaks and valleys situated above the top surface flattened or plastically deformed at a higher temperature.

**Figure 24:**
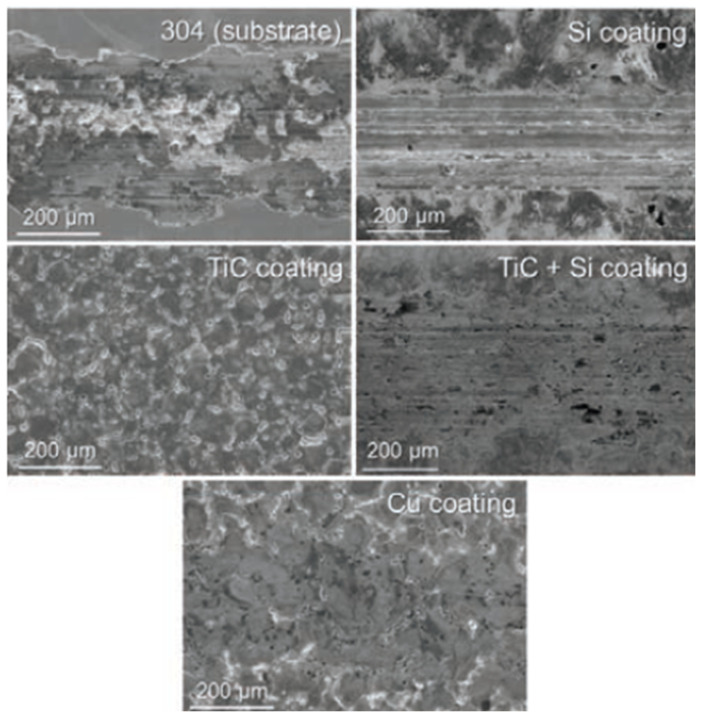
SEM images showing the wear tracks of 304 stainless steel coated by Si, TiC, TiC + Si, and Cu [132]. (With kind permission from Elsevier).

Murray and Clare [132] investigated the surface of 304 stainless steel coated by the EDC process. A ball-on-flat linear reciprocating wear test was conducted to examine the wear resistance and coefficient of friction. A single TiC-coated layer provided the lowest coefficient of friction, with no wear track on the coated surface. The surface modified by the conventional EDM process using a Cu electrode showed a better tribological performance than the Si-coated layer surface produced by the EDC method. Wear tracks were clearly visible from the SEM images of Si-coated surface, shown in Figure 24. A multilayer coating of TiC and Si showed lower surface defects; however, the tribological performance was not better than the single TiC-coated layer. A lower hardness of Si resulted in the removal of coatings from the multilayer coated surface.

## 6. EDM-Based Novel Techniques of Surface Modification and Applications

**Figure 25:**
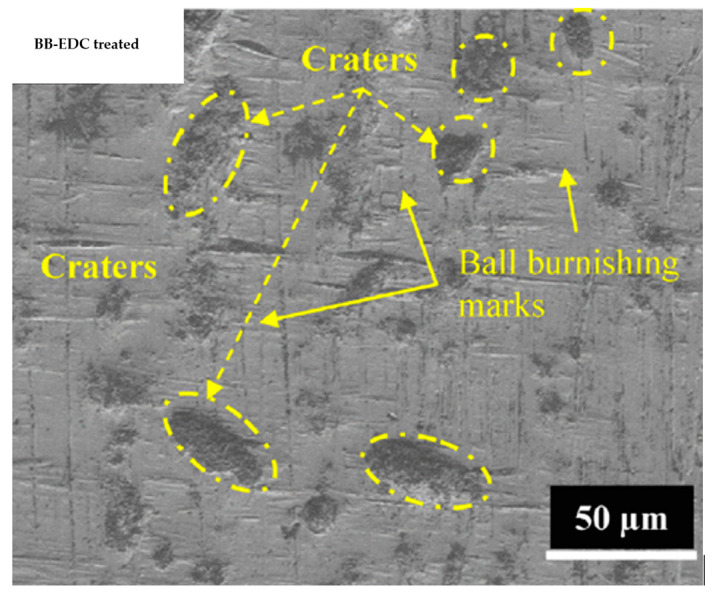
SEM micrograph of the BB-EDC treated β-phase Ti alloy surface [133]. (With kind permission from Elsevier).

Wandra et al. [133] modified the surface of new class β-phase Ti alloy (Ti-Nb-Ta-Zr) by conventional EDM and ball burnishing-assisted electrical discharge cladding (BB-EDC) process. This alloy has widespread applications in the biomedical industry. Hydroxyapatite powder was used with the dielectric in the case of the BB-EDC processing. The EDMed surface consisted of severe surface defects along with high ridges of deposited metals. Furthermore, a higher surface roughness (2.61 µm) and craters were observed, which can introduce corrosion and affect the fatigue performance of the implants. Ca, P, and O were deposited over the newly formed surface along with the reinforcement of HAp in the case of the BB-EDC process. The use of ZrO_2_ balls resulted in the flattening of the high ridges of deposited metals, which is shown in Figure 25. As a result, the BB-EDC-treated surface was smoother with less craters compared with the EDMed surface. The surface roughness was significantly decreased (1.16 µm) compared with the EDMed and untreated surface.

Tyagi et al. [134] applied EDC onto the surface of AISI mild steel by using a green compact tool of Cu + MoS2 + WS2 + hBN powder. The copper powder was used as the binder of the compact electrode. The specific wear rate was measured by varying the loads, such as, 5 N, 10 N, and 20 N. The result of the wear test showed that the wear rate was lower when the load was in a range of 5–10 N compared with the substrate. The wear rate sharply accelerated with the increase in load from 10 N to 20 N, which was similar to the substrate. As a result, the coating above the AISI steel surface was easily worn out. However, the coating displayed tremendous resistance against wear and friction compared with the mild steel. Moreover, this hierarchical coating increased the water contact angle and turned the surface to a super-hydrophobic state (152.2°).

Maideen et al. [135] modified the surface of Al 7075 by mixing Mo and Ni powder particles in one case and Mo and W powders in another case with the dielectric medium. The successful deposition of powder particles was obtained in the newly formed layer in each case. A better surface was achieved in the case of Mo and Ni when the peak current, pulse on-time, and pulse off-time were 6 A, 100 µs, and 150 µs, respectively. On the other hand, a smooth surface finish was achieved for Mo and W when 6 A, 100 µs pulse on-time, and 316.58 µs pulse off-time were considered.

**Figure 26:**
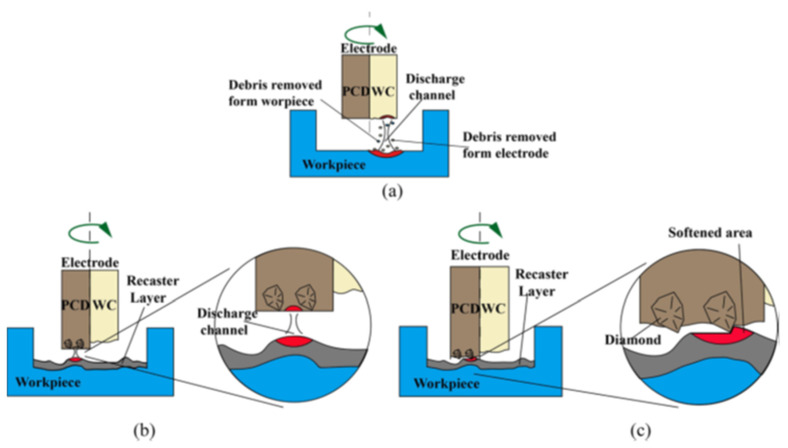
(**a**–**c**) Alternating energy electrical discharge machining (AE-EDM) process [136]. (With kind permission from Elsevier).

Wang et al. [136] used alternating energy electrical discharge machining (AE-EDM) to modify the surface of Ti-6Al-4V by tungsten carbide–polycrystalline diamond (WC-PCD) electrode. The AE-EDM process steps are shown in Figure 26. This new method in addition to micro-grinding prevented a portion of molten metal from being re-solidified over the recast layer. As a result, the newly formed layer in the case of AE-EDM was found to be thinner compared with the recast layer produced by the conventional EDM process. The surface microhardness increased by 25.6% in the AE-EDM process compared with the traditional EDM process using Cu as the tool electrode, mainly because of the deposition of WC in the AE-EDM process.

**Figure 27:**
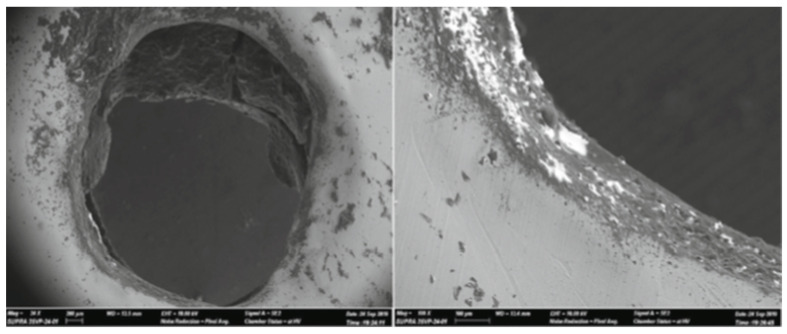
SEM images showing micro-holes produced in AlN by the micro-EDM process [137]. (With kind permission from Springer).

Rashid et al. [137] investigated the machining of an electrically non-conductive aluminum nitride (AlN) ceramic surface by using micro-EDM. It was found that the material was removed due to the combination of melting, evaporation, and thermal spalling. The electrical conductivity to the machined zone was introduced by the carbon deposition on the wall of the workpiece. The plasma channel radius was reduced in this experiment, and the energy was therefore concentrated when it passed from the tool to the workpiece. The SEM images shown in Figure 27 indicate that a rough surface was produced even when lower discharge energy was involved in this process.

## 7. Future Research Directions

Regardless of the many merits of EDM and EDC, these processes are not completely eco-friendly. The used wires in the case of wire-EDM, in other words, the byproducts produced during the EDM process, can be a threat to green manufacturing technology. Thus, research ought to be conducted to make this non-conventional machining process more environmentally friendly; the reuse of the byproducts can be a solution to that. Sustainable EDM process will be at the forefront of the future research directions in the area of EDM. Sustainable manufacturing has been at the core of research interests for the last decade. Although many studies have focused on improving the sustainability aspects of the conventional mechanical machining processes, very little research has focused on improving the sustainability aspects of the EDM process. The sustainability aspects should not only consider the reduction of waste, cost, and energy, but also focus on making the machined surfaces more functional so that secondary or post-processing can be eliminated or minimized.

The deposition of oxygen and carbon on the machined surface during the EDM process is an unavoidable phenomenon. Although much research has aimed to use the deposition of oxides and carbides in a favorable way by making targeted and controlled surface modification, very little research has focused on minimizing the deposition of oxide and carbide formation on the surface. Oxide formations were found to be beneficial for biomedical applications, and carbide formations were found to be beneficial for tribological applications, although the vice-versa is not true.

Therefore, future research should focus on controlling any unwanted material deposition on the surface by innovating new variations of EDM processes.

**Figure 28:**
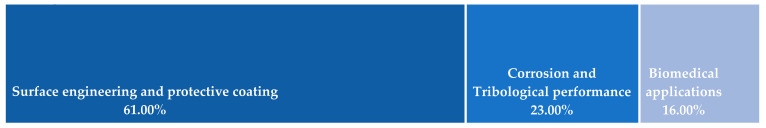
Contribution of EDM in different applications (found in different publications reviewed in this study).

Figure 28 shows the percentage contribution of EDM in different applications that were found in various publications while reviewing papers for this study. It is clear that many research works have been conducted on generating a protective coating over the surface and surface engineering, whereas comparatively, few works have been conducted to examine the tribological and biomedical performance of the EDMed surface. So, these mentioned areas can be explored more in future research works.

The electro-discharge coating (EDC) process can work as an independent process with targeted applications. The EDC process can eliminate the need for post-process coating, thus reducing the cost associated with the coating process. This area has the potential to be explored for more innovative applications of EDM-coated surfaces.

One of the novel techniques of electro-discharge coating is the use of composite electrodes to modify the surface with a targeted deposition of composite elements. In many cases, the rate of electrode wear for these composite electrodes are too high and difficult to control. Future research should focus on a sensor-based or machine learning-based control of wear rate from the composite electrodes for the controlled surface modification on the EDMed surfaces.

One important shortcoming of the modified surface after EDM is its poor fatigue performance. This is associated with the formation of craters, pores, and other defects at the surface and sub-surface of machined parts. There have been some research studies on minimizing the recast layer thickness, thus minimizing the number and intensity of defects and improving fatigue strength. Future research should consider surface modification for improving fatigue strength, possibly by adding specific elements on the machined surface, which could improve fatigue performance.

It was found from various studies that a handsome amount of time is required to generate a smoother surface or coating, which is a drawback of the EDM or EDC process. Future research can also be directed to generate smoother or optimum coating by investing less time. Hybrid machining processes combining traditional or other non-traditional manufacturing processes with EDM can resolve this issue and improve machining time. Hybrid processes allow two physical principles to work synchronously or sequentially to improve the machining time or other machining parameters. A common example of a hybrid process that was found to improve machining time is vibration-assisted EDM. Researchers should focus on the process development of innovative hybrid processes that has not been reported yet.

Another popular process of targeted surface modification using the EDM process is the addition of powder containing target elements that need to be deposited on the machined surface. Various powders have been used in PMEDM processes for targeted surface modification. However, the use of powders such as HAp, B_3_N, MoS_2_, W, etc., should be studied in detail to better understand the effect of these powders in the surface modification and phase changes of materials and alloys.

**Figure 29:**
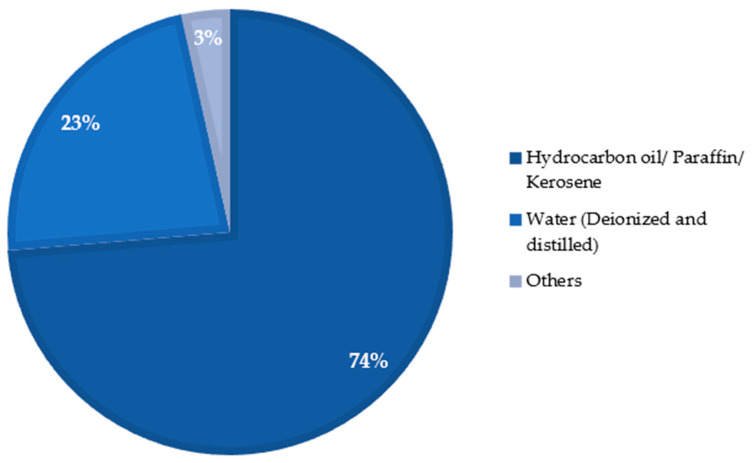
Types of dielectric fluids used in EDM processes (found in different publications reviewed in this study).

The dielectric fluid used in EDM plays a major role in surface modification as the dielectric fluids break down during EDM because of the high temperature generated at the tool–workpiece gap and because the decomposed hydrocarbon materials from the dielectric are deposited on the machined surface. From Figure 29, it is clear that EDM oil and deionized water are the two common dielectric liquids used in EDM, although other dielectric media, such as nitrogen gas, air, silicon oil, etc. were found to work as well. Future research should also focus on using innovative dielectric media, whether fluid or gaseous in nature, for targeted surface modification. The application of some gaseous media as dielectric oil may also reduce the deposition of carbon on the machined surface if carbon deposition on the EDMed surface is unwanted for a specific application.

The surface modification during micro-EDM has a great potential to be used as a coating technique for miniaturized parts and components. The surface engineering at the micro-scale has not been explored much in the literature. Future research should consider exploring various aspects of micro-EDM and micro-EDM-based hybrid processes for the protective coating of miniaturized parts. Micro-EDM is used for fabricating miniaturized parts for aerospace, automotive, and biomedical applications, such as fuel injector, micro-needles, micro-tools, etc. The spontaneous coating generated during the micro-EDM process can get rid of additional post-processing steps for coating the micro parts. Providing coating on the micro-scale parts are always challenging because of the risk of damaging the micro-features on the parts. The coating process while machining the same parts can solve the problem by eliminating the risk of damaging the part in the subsequent coating process. Similarly, corrosion resistance, wear resistance, improved hardness, etc. can be incorporated into the part when being machined by the micro-EDM process by carefully designing some of the electro-discharge coating techniques discussed in this paper.

## 8. Conclusions

The nature of the modified surface in the EDM process plays a vital role in deciding its application in various industrial sectors. Typically, the surfaces machined or modified by EDM and EDC have a higher surface micro-hardness and better tribological properties. It can be seen from the extensive literature review presented in this study that the surface can be modified or tailored with the target elements or compound on the surface that is machined by EDM. In some cases, the EDM process can provide better and more cost-effective surface treatments in contrast to physical vapor deposition (PVD), chemical vapor deposition (CVD), microarc oxidation (MAO) coating, or plasma electrolytic coating, and so on. Noteworthy progress has been made in the field of using EDM as a surface modification technique for functional applications, such as a lubricating surface, improved wear and corrosion resistance, improved biocompatibility, etc. Nonetheless, future research should focus on identifying innovative applications of the machined surfaces modified by the various EDM-based hybrid machining processes. Bringing the technology from the lab to real-life applications will be the goal for future researchers working on applying EDM for targeted surface modification for functional applications.

## Figures and Tables

**Table 1:** Generation of layers over the base material by different EDM processes.

Authors	EDM Type	Workpiece	Tool Electrode	Dielectric	Powder	Elements/Compounds in Newly Formed Layer
Mohri et al. [70]	DSEDM	C steel	Al green compact electrode	Hydrocarbon oil	-	Fe_3_C, AlFe_3_C_0.5_, α-Fe
Shunmugam et al. [71]	DSEDM	HSS	WC-Fe P/M electrode	Kerosene	-	WC, W_2_C, FeC, (Fe_3_C)H
Wang et al. [20]	DSEDM	C steel	Ti powder green compact electrode	Hydrocarbon oil	-	TiC, Fe
Ablyaz et al. [51]	DSEDM	Duplex SS	Graphite, Cu-W, W	EDM oil	-	O, oxide, tungsten carbides
Tsai et al. [64]	DSEDM	AISI 1045 medium C steel	Cu-Cr, Cu	Kerosene	-	Cu, Cr
Simao et al. [72]	DSEDM	AISI H13 steel	WC/Co partially sintered electrode	Hydrocarbon oil	-	WC
Patowari et al. [66]	DSEDM	C-40 grade steel	W-Cu	EDM oil grade 30	-	W, W_2_C, Cu, Fe
Senthilkumar et al. [65]	DSEDM	Mild steel	Cu-40% B_4_C	Hydrocarbon oil	-	B_4_C, BFe_2_, CuB_28_, FeCu_4_
Sidhom et al. [52]	DSEDM	316L SS	Graphite	Paraffin oil	-	Cr_7_C_3_
Patowari et al. [67]	DSEDM	C-40 grade steel	WC-Cu	EDM oil	-	WC, W_2_C, Cu, Fe
Afzaal Ahmed [29]	DSEDM	Al	Ti + B_4_C + Al P/M electrode	Hydrocarbon oil	-	AlB_2_, TiC, AlTi_3_, TiB_2_, Al_4_C_3_
Mehmood et al. [49]	DSEDM	Al 2024 T6	Cu	Kerosene	-	C
Arooj et al. [48]	DSEDM	Al 6061 T6	Cu	Kerosene	-	Cu, O, C
Samrah et al. [69]	DSEDM	Al 7075 alloy	Inconel 718 + Al green compact electrode	Hydrocarbon oil	-	Al, Al_3_Ni, NbNi_3_, Fe_5_C_2_
Hwang et al. [73]	DSEDM	Ni	Ti + Gr multilayer electrode	SE fluid 180	-	TIC, C
Li et al. [68]	DSEDM	Ti-6Al-4V	Cu-SiC	EDM oil	-	Cu, Si, C, TiC, TiSi_2_
Beri et al. [74]	DSEDM	Inconel 718	Cu-W P/M electrode	EDM oil	-	Fe_6_W_6_C, Cr_2_F_14_C, N_2_Mo_4_C
Klocke et al. [75]	WEDM	Steel Vanadis 4 Extra	Brass wire	Hydrocarbon oil	-	Fe, Cu, Cr, Zn, Mo, V
Kumar et al. [76]	WEDM	Pure Ti	Brass wire	DI water	-	TiO_2_, TiO_0.325_, Ti_2_O_3_, Cu_3_TiO_4_, Ti_3_ZnC, Zn_2_Ti_4_C, TiC
Mahbub et al. [43]	WEDM	Ti-6Al-4V	Brass wire	DI water	-	Cu, Zn, TiO_2_
Shinonaga et al. [77]	WEDM	Ti-6Al-4V	Brass wire	DI water	-	Ti_2_O_3_, TiO, Ti
Rahman et al. [78]	WEDM	Ti-6Al-4V	Mo wire	DI water in oil emulsion	-	Al(OH)_3_, V_2_O_5_, rutile
Bonny et al. [79]	WEDM	ZrO_2_-TiCN	Brass wire	DI water	-	ZrTiO_4_, ZrO_2_
Molinetti et al. [80]	PMEDM	AISI H13 steel	Cu	Hydrocarbon oil	Mn, Si	SiC, FeSi, Mn_4_C, Mn_4_C_2_
Khan et al. [81]	PMEDM	Mild steel	Cu-W	Kerosene	Al_2_O_3_, TiC	Cu, W, Al, C
Ekmekci and Ersoz [82]	PMEDM	IF steel	Cu	Tap water, oil	SiC	SiC, α-Fe, ɣ-Fe
Yan et al. [23]	PMEDM	Pure Ti	Cu	Distilled water	Urea	TiN
Devgan and Sidhu [83]	PMEDM	β-Ti	Graphite	DI water	MWCNT	TiC_2_, TiO, Ti_2_O_3_, Ti_3_O_5_, Nb_2_O_5_, TiH, ZrO_2_, ZrC, Nb_2_C
Chen et al. [84]	PMEDM	Grade 4 pure Ti	Grade 4 pure Ti	DI water	Ti	α-Ti, TiO
Tijo et al. [85]	PMEDM	Ti-6Al-4V	Cu	Kerosene	Ti, B_4_C	TiB_2_, TiB, TiC, TiO_2_, Ti, C
Bains et al. [86]	PMEDM	Ti-6Al-4V	Cu	EDM oil	n-HA	TiC, TiO_2_, VSi_2_, Ca_3_(PO_4_)_2_, P, CaTiO_3_
Janmanee and Muttamara [87]	PMEDM	WC90-Co10	Cu	Shell EDM Fluid 2A	Ti	TiC
Hu et al. [53]	PMEDM	SiCp/Al	Cu	Kerosene	Al	C, Al, Si, SiC
Singh et al. [58]	PMEDM	Super Co 605	Graphite	EDM oil	Graphite	C
Sharma et al. [59]	PMEDM	Mg-4Zn	Cu	EDM oil	Zr, Mn	Carbides of powder elements
Abdu Aliyu et al. [88]	PMEDM	Zr-based BMG	Pure Ti	Hydrocarbon oil	HA	ZrC, TiC, CaTiO_3_
Jahan et al. [30]	µ-EDM	NiTi	WC	Commercial EDM oil	-	NiTiO_3_, W
Jahan et al. [62]	Milling µ-EDM	Tungsten carbide	W	EDM oil	Graphite	C
Davis et al. [63]	PM µ-EDM	Ni55.6Ti44.4	Cu, Brass	EDM oil	Zn	Metal oxides and carbides
Jahan et al. [61]	DS µ-EDM	Tungsten carbide	W, CuW, AgW	EDM oil	-	C
Mohanty et al. [89]	PM µ-EDM	Ti-6Al-4V	Brass	DI water	hBN	Ti, TiAlN, TiN, Al_2_O_3_, BN, ZnO, CuO, TiO_2_

**Table 2:** Recent works conducted in the biomedical sector using EDM processing.

EDM Type	Workpiece	Powder	Remarks
Conventional EDM [122]	Fe-Al-Mn Alloy	-	Resulted in improved biocompatibility and osseointegration, more cell attachment
Conventional EDM [123]	Ti-6Al-4V	-	Osteoblastic cells completely spread on the EDMed surface. Noticeable MG-63 cells attachment and proliferation confirmed its usability for clinical purposes.
Conventional EDM [126]	Co-Cr and Ti	-	Ti electrode was better compared with the Cu electrode in manufacturing Co-Cr and Ti dental alloys.
DSEDM [117]	Ti_50_Ni_50_, Ti_50_Ni_49.5_Mo_0.5_ and Ti30Nb1Fe1Hf	-	Defects on the recast layer were insignificant. TiO was found on TNM and TNB. Surface roughness was favorable for oral implants.
WEDM [43]	Ti-6Al-4V	-	Presence of β and α + β helped with cell attachment. A TiO layer formed, which prevented the implant from being corroded, thus resulting in a better biofunctionality.
µ-EDM [30]	Ti-6Al-4V and NiTi	-	TiO_2_ and WO_2_ layers formed in the case of Ti-6Al-4V alloy and NiTiO_3_ film formed over NiTi. These layers resulted in an improved biocompatibility.
PMEDM [59]	Mg/Zn	Zr, Mg	Biocompatibility and corrosion resistance was better when Zr powder was used.
PMEDM [127]	316L steel	Hap	A thin coating formed on the specimen. The authors obtained 70% of living cells, which indicated an improved cell proliferation as well as biocompatibility.
PM-µEDM [129]	Mg alloy AZ91D	Hap	A glossy recast layer was formed, and an increased hydrophobicity was achieved. As a result, the modified surface can be used in medical sectors.
PM-µEDM [63]	Ni_55.6_Ti_44.4_	Zn	Higher cell viability percentage ensured a tremendous role of the modified alloy in the broken tissue recovery, and this modified alloy is suitable for cardiovascular applications.

## Data Availability

Data is available upon request.
